# Demographic History of Indigenous Populations in Mesoamerica Based on mtDNA Sequence Data

**DOI:** 10.1371/journal.pone.0131791

**Published:** 2015-08-20

**Authors:** Antonio González-Martín, Amaya Gorostiza, Lucía Regalado-Liu, Sergio Arroyo-Peña, Sergio Tirado, Ismael Nuño-Arana, Rodrigo Rubi-Castellanos, Karla Sandoval, Michael D. Coble, Héctor Rangel-Villalobos

**Affiliations:** 1 Departamento de Zoología y Antropología Física, Facultad de Biología, Universidad Complutense de Madrid, Madrid, Spain; 2 Laboratorio de Identificación Genética, GENOMICA S.A.U., Grupo Zeltia, Parque Empresarial Alvento, Calle Vía de los Poblados 1, Edificio B 1ª Planta, 28033, Madrid, Spain; 3 Instituto de Investigación en Genética Molecular, Centro Universitario de la Ciénega, Universidad de Guadalajara, Ocotlán, Jalisco, Mexico; 4 Laboratorio de Genética, Centro de Investigaciones Regionales Hideyo Noguchi, Universidad Autónoma de Yucatán (UADY), Mérida, Yucatán, Mexico; 5 Department of Genetics, Stanford University School of Medicine, Stanford, California, United States of America; 6 American Registry of Pathology, Armed Forces DNA Identification Laboratory, Dover, Delaware, United States of America; University of Perugia, ITALY

## Abstract

The genetic characterization of Native American groups provides insights into their history and demographic events. We sequenced the mitochondrial D-loop region (control region) of 520 samples from eight Mexican indigenous groups. In addition to an analysis of the genetic diversity, structure and genetic relationship between 28 Native American populations, we applied Bayesian skyline methodology for a deeper insight into the history of Mesoamerica. AMOVA tests applying cultural, linguistic and geographic criteria were performed. MDS plots showed a central cluster of Oaxaca and Maya populations, whereas those from the North and West were located on the periphery. Demographic reconstruction indicates higher values of the effective number of breeding females (Nef) in Central Mesoamerica during the Preclassic period, whereas this pattern moves toward the Classic period for groups in the North and West. Conversely, Nef minimum values are distributed either in the Lithic period (i.e. founder effects) or in recent periods (i.e. population declines). The Mesomerican regions showed differences in population fluctuation as indicated by the maximum Inter-Generational Rate (IGRmax): i) Center-South from the lithic period until the Preclassic; ii) West from the beginning of the Preclassic period until early Classic; iii) North characterized by a wide range of temporal variation from the Lithic to the Preclassic. Our findings are consistent with the genetic variations observed between central, South and Southeast Mesoamerica and the North-West region that are related to differences in genetic drift, structure, and temporal survival strategies (agriculture *versus* hunter-gathering, respectively). Interestingly, although the European contact had a major negative demographic impact, we detect a previous decline in Mesoamerica that had begun a few hundred years before.

## Introduction

The peopling of the New World is still a controversial debate today. In fact, there is no consensus on whether the first settlers arrived in one [1,2] or several migration waves [3, 4]. Nevertheless, most recent research based on genetic data supports the view that the New World was peopled across the Bering Strait about 18,000 years before the present (ybp) [5]. Some authors propose two routes for a rapid human expansion across the continent, one coastal and the other inland; although other alternative routes have been proposed [6–8]. The hypothesis for the human colonization of America is largely based on the study of mitochondrial DNA (mtDNA) which, from the very first studies, has shown that extant Native American populations exhibit six mtDNA different haplogroups (hgs). These can be classified into the following autochthonous hgs: A2, B2, C1, D1, D4h3, and X2a [9]. The fact that these lineages are restricted to a specific geographical area allows us to reconstruct the micro-evolutionary history of Mesoamerica and bordering areas.

Current mtDNA studies in the new continent have focused on two new lines of research. On the one hand, the genetic characterization of skeletal remains using ancient DNA techniques provides information on the composition and genetic structure of pre-Columbian populations [10–13]. On the other hand, the complete sequencing of mitochondrial genomes allows for a greater analytical and interpretative depth of genetic information [14–17].

According to Kirchhoff [18], Mesoamerica is defined as neither a geographic region nor a socio-political unit, but rather, as an area occupied by populations that share cultural characteristics. It is also considered that at its peak the northern limit overlapped with the southern frontier of southwestern North America [19, 20].

Considering cultural, geographical and historical criteria, Mesoamerica can be divided into different cultural areas [21–24]. In this paper seven clusters have been considered [25–32] in line with those used by other authors to reconstruct the history of Mesoamerica [33,34]. The peoples who share these areas have common elements although they do not necessarily constitute a single ethnic group, in fact, in many cases, they do not even share the same language. Within these areas, and even between them, interactions and migrations occurred as a consequence of geographical proximity [35, 36], trade [37, 38] and pre-hispanic politics [39, 40].

In this paper these seven regions have been used to present a systematic classification of the results and as a starting point to explore the possible existence of genetic structure in the region.

It is necessary to clarify that, although the work is focused on Mesoamerica, some northern populations come from Aridoamerica. This is an arid region north of Mesoamerica covering a territory that is distributed between northern Mexico and southern USA, traditionally inhabited by nomadic or seminomadic peoples [27].

Another important aspect concerning Mesoamerica is its chronological classification according to archaeological data. In brief, it can be classified into five periods [19,23,41]. The chronology begins with the archaic period or lithic stage (15000–2500 ybp) characterized by the first evidence of a human presence. In the Preclassic period between 2500 ybp-150/200 years after present (yap), the settling of Mesoamerica gathered pace and the use of agriculture and pottery began. In the immediate aftermath, or Classic (150 / 200–900 yap) period, intensive agriculture was developed and an increase in the number of large population centers is detected. In the following period, known as Postclassic (900–1521 yap), numerous movements of population occur accompanied by a wide diffusion of cultural elements. This period ends with the arrival of the Europeans and the beginning of the colonial period (1951 yap-present) [42,43].

Some of the most representative American cultures flourished in Mesoamerica, for example the Aztecs and Mayas, in addition to numerous indigenous groups, some of whom are the descendants of those ancient cultures. These populations emerged in a homogeneous pattern of rituals, politics and architecture, and shared a similar lifestyle based upon agriculture, as well as a similar social and commercial organization. This relative cultural homogeneity is also supported by the archaeological and anthropological data [32].

Studies based on the genetic variations in native Mexican populations demonstrate that there is a high degree of differentiation between populations and a paternal heterogeneity correlated with geography [33,44]. However, the maternal lineages do not display significant population structure in relation to linguistic criteria, suggesting that genetic divergence predates linguistic diversification in Mexico [45].

In recent years, great efforts have been made to reconstruct the history and genetic relationships between human groups [46]. Among these advances stands out the development of software that makes it possible to reconstruct human population history using mtDNA sequences [17]. In the present work, we studied the genetic structure, diversity and genetic relationship of some indigenous groups from Mesoamerica applying the Bayesian skyline methodology [47]. We aimed to reconstruct the genetic and demographic history of these groups in the context of other indigenous peoples inhabiting the neighboring regions. For this purpose, the mitochondrial D-loop region of 520 samples from eight Mexican indigenous groups was sequenced (five from the Maya region and three from the West of Mexico). For a deeper insight into Mesoamerican history, the results have been compared with other populations from Mesoamerica and Aridoamerica. The 28 native Mexican populations represent 19 indigenous groups and five of the seven Mesoamerican cultural areas, including the Aridoamerican region.

## Material and Methods

### Population sampling

**Fig 1:**
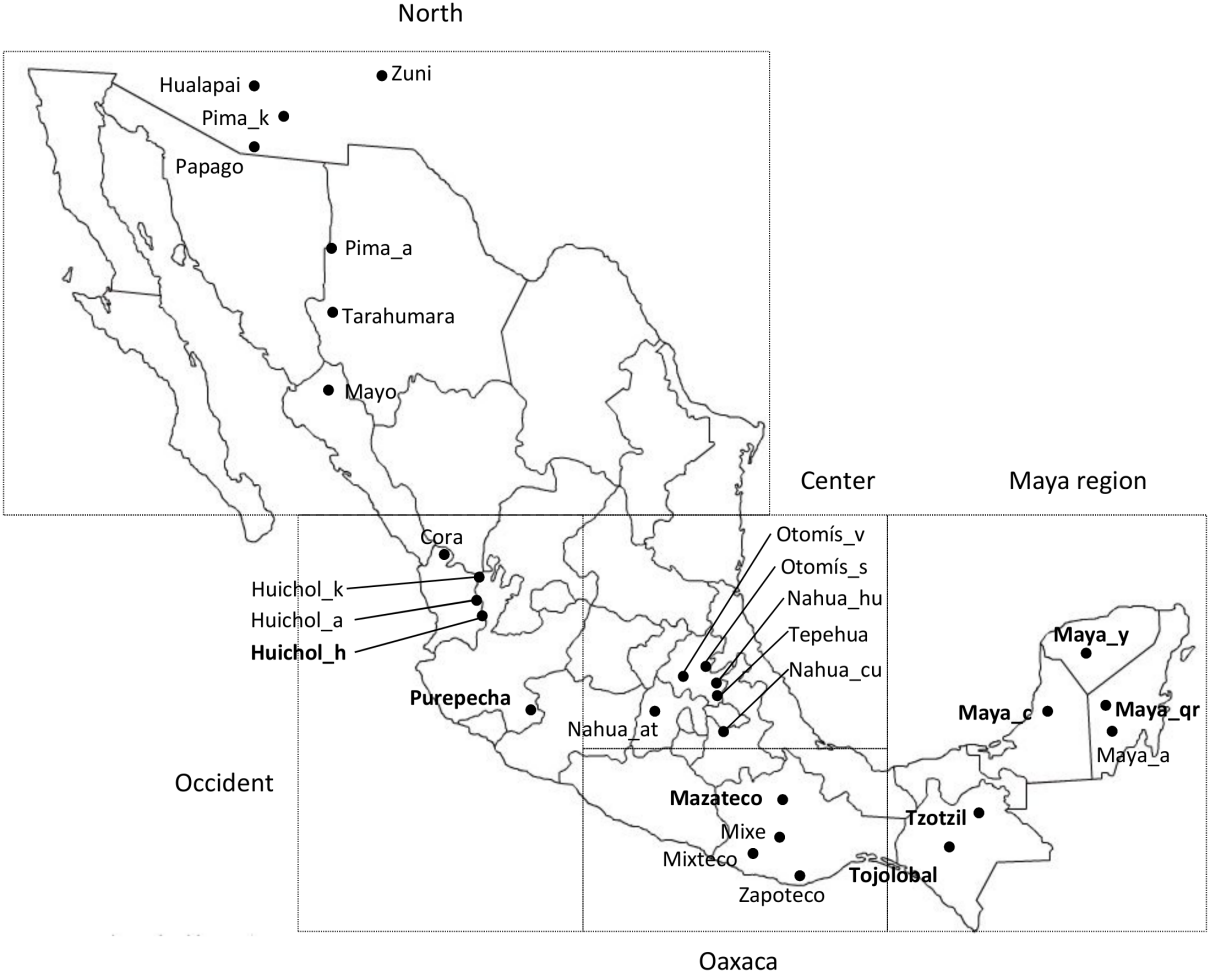
Geographical location of the 28 populations included in the study. Quadrants represent the regions zoomed in the supplementary information based on the cultural areas of Mesoamerica. Populations studied in this paper appear in bold. For abbreviations please check Table 1.

The mtDNA control region of 520 individuals representing eight Mexican indigenous groups was sequenced. Five of the groups were of Maya filiation: Yucatan Maya (n = 40), Quintana Roo Maya (n = 74), Campeche Maya (n = 37), as well as Tojolabal (n = 74) and Tzotzil (n = 87) groups from Chiapas. The other three indigenous groups are Mazateco from Oaxaca (n = 41), Purepecha from Michoacán (n = 65), and Huichol from Nayarit (n = 102) (Table 1, Fig 1).

**Table 1:** Geographical and cultural information from the 28 Native American populations included in this study.

Name	Acronym	n	Indigenous group	Language	Cultural area/ Region	Ref
**Zuni**	Zuni	50	Zuni	Azteca-Tanoan	Aridoamerica	44
**Hualapai**	Hualapai	76	Hualapai	Cochimí-Yuman	Aridoamerica	44
Pima* (Akimel O’odham)	Pima_k	98	Pima	Uto-Aztecan	Aridoamerica	44
Papago* (Tohono O’odham)	Papago	42	Papago	Uto-Aztecan	Aridoamerica	44
Pima* (Akimel O’odham)	Pima_a	49	Pima	Uto-Aztecan	Aridoamerica	67
**Tarahumara**	Tarahumara	73	Tarahumara	Uto-Aztecan	North/Mesoamerica	44
**Mayo**	Mayo	55	Mayo	Uto-Aztecan	North/Mesoamerica	67
**Cora**	Cora	72	Cora	Uto-Aztecan	West/Mesoamerica	44
**Huichol**	Huichol_k	62	Huichol	Yuto-Nahua	West/Mesoamerica	44
**Huichol**	Huichol_a	36	Huichol	Yuto-Nahua	West/Mesoamerica	67
**Huichol**	Huichol_h	102	Huichol	Yuto-Nahua	West/Mesoamerica	This study
**Purepecha**	Purepecha	65	Purepecha	Tarasco	West/Mesoamerica	This study
**Otomí valle**	Otomi_v	81	Otomí	Oto-Manguean	Center/Mesoamerica	67
**Otomí sierra**	Otomi_s	90	Otomí	Oto-Manguean	Center/Mesoamerica	67
**Nahuas Huasteca**	Nahua_hu	189	Nahua	Uto-Aztecan	Center/Mesoamerica	67
**Nahuas Cuetzalan**	Nahua_cu	46	Nahua	Uto-Aztecan	Center/Mesoamerica	44
**Nahuas Actopan**	Nahua_at	50	Nahua	Uto-Aztecan	Center/Mesoamerica	44
**Tepehua**	Tepehua	51	Tepehua	Totonacan	Center/Mesoamerica	67
**Mazateco**	Mazateco	41	Mazateco	Oto-Manguean	Oaxaca/Mesoamerica	This study
**Mixe**	Mixe	52	Mixe	Mixe-Zoquean	Oaxaca/Mesoamerica	44
**Mixteco**	Mixteco	67	Mixteco	Oto-Manguean	Oaxaca/Mesoamerica	44
**Zapoteco**	Zapoteco	85	Zapoteco	Oto-Manguean	Oaxaca/Mesoamerica	44
**Maya Yucatan**	Maya_y	40	Maya	Mayan	Maya/Mesoamerica	This study
**Maya Campeche**	Maya_c	37	Maya	Mayan	Maya/Mesoamerica	This study
**Maya Quintana Roo**	Maya_qr	74	Maya	Mayan	Maya/Mesoamerica	This study
**Maya**	Maya_a	44	Maya	Mayan	Maya/Mesoamerica	67
**Tzotzile**	Tzotzil	87	Maya	Mayan	Maya/Mesoamerica	This study
**Tololabales**	Tojolabal	74	Maya	Mayan	Maya/Mesoamerica	This study

Throughout the text, different synonymous terms are used to refer to the populations studied. Native American refers to native populations but not necessarily from Mesoamerica. The terms native and indigenous are used interchangeably to refer to the Amerindian populations. Moreover, Native Mexican includes populations currently distributed in Mexican territory which may belong to either Mesoamerica or Aridoamerica. Whenever we refer to mestizo-and therefore non-indigenous- populations, this is specified in the text.

DNA was extracted from fresh-blood samples by means of the salting-out method. Prior to their inclusion in our study, all volunteers signed an informed consent form according to the ethical guidelines of the Helsinki Declaration. This work was approved by the Research Ethics Committee of the CUCiénega, Universidad de Guadalajara, as part of the project “Comprehensive approach of genetic anthropology of the Mexican populations based on mtDNA, STRs and Y-chromosome” (CONACyT-Mexico, grant N° 129693).

### Mitochondrial DNA sequencing

The control region for all 520 samples was amplified [48] using the high-throughput strategy [49]. For the PCR amplification of the full control region, we used a single primer pair (F15971/R599), or three overlapping primer pairs (F15878/R16410; F16190/R285; and F15/R649) when the full control region amplification was initially unsuccessful. Primer sequences for each sequencing primer are available in the specialized bibliography [50,51]. Post-PCR deactivation of unincorporated oligonucleotides and dNTPs were treated enzymatically with ExoSAPIt (USB, Cleveland, OH, USA). Cycle sequencing of the PCR amplicons was performed^40^ using Big Dye version 1.1 of ThermoFisher (previously Life Technologies/Applied Biosystems, Foster City, CA, USA). Unincorporated fluorescent ddNTPs were removed with the Performa V3 96-well short plate (Edge Biosystems, Gaithersburg, MD, USA). Sequence data were generated on either the Applied Biosystems 3130*xl* using POP-6 polymer or the 3730 Genetic Analyzer using POP-7 polymer. Sequences were verified by two different analysts and an additional quality control check was performed by EMPOP [52]

### Data analysis

The mtDNA sequences were aligned with Sequencher version 4.8 (GeneCodes, Ann Arbor, MI). For diagnostic purposes, hgs were identified based on polymorphic sites and using different online resources (MitoTool, http://www.mitotool.org/index.html); absolute frequencies were obtained by means of the gene counting method (Table 2). Statistical analyses were carried out using DNAsp and Arlequin v3.1, including different diversity estimators [53,54]: haplotype number (k), polymorphic sites (s), haplotype diversity (Ĥ), nucleotide diversity (π), average pairwise differences (θ) and tests of neutral selection such as Tajima´D (D) and Fu´s test (FS). The genetic relationships between the eight study populations were evaluated by means of the pairwise differences (Table A in S1 File) and F_ST_ genetic distances Analyses of Molecular Variance (AMOVAs) applying different population clustering criteria were also performed, as indicated in the tables of the results section (Table B in S1 File).

**Table 2:** Hgs frequencies (%) for the eight Native Mexican populations according to the complete mtDNA control region.

	Maya_qr	Maya_y	Maya_c	Tzotzil	Tojolabal	Mazateco	Purepecha	Huichol_h	Total
	n = 74	n = 40	n = 37	n = 87	n = 74	n = 41	n = 65	n = 102	n = 520
**A2**	64.9	65.0	70.3	41.4	25.7	53.7	83.1	25.5	49.4
**B2**	16.2	12.5	5.4	24.1	58.1	34.1	1.5	27.5	24.2
**B4b1**	0.0	2.5	0.0	0.0	0.0	0.0	0.0	0.0	0.2
**C1**	14.9	12.5	18.9	25.3	0.0	2.4	10.8	47.1	19.4
**D1**	4.1	7.5	5.4	0.0	16.2	0.0	4.6	0.0	4.4
**D4h3a**	0.0	0.0	0.0	9.2	0.0	9.8	0.0	0.0	2.3

**Fig 2:**
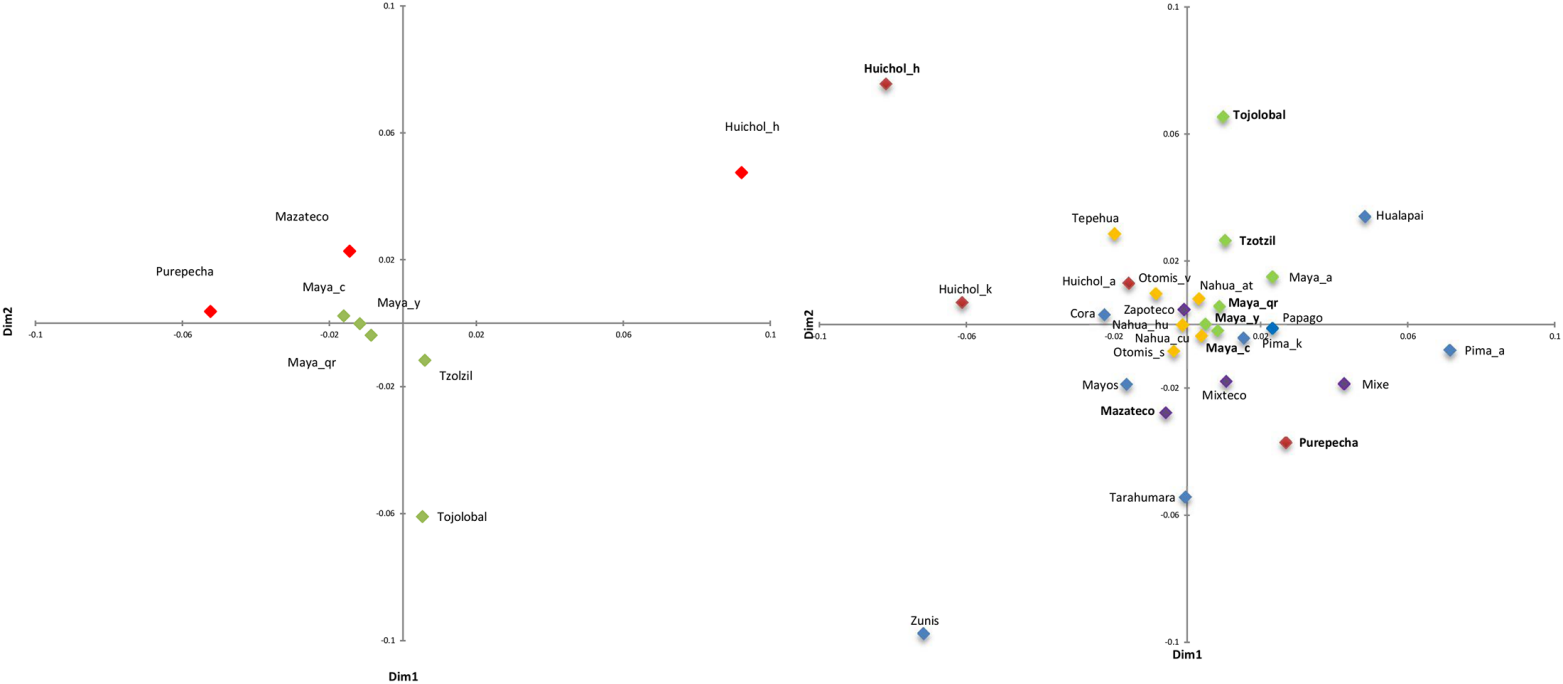
MDS plot representing Fst distances between Native American groups based on mtDNA D-loop region. Bold letters denote the indigenous groups studied herein. a) MDS plot for eight Native Mexican groups; b) MDS plot for 28 Native American groups. Colors indicate the cultural regions to which the populations belong: green, Maya region; blue, North; red, West; yellow, Center; and purple, Oaxaca. Bold letters indicate Mexican indigenous groups included in this study.

The results were compared with 20 populations from Mesoamerica or nearby regions (Table 1, Fig 1). The global study for the 28 populations was based on the F_ST_ calculation (Table C in S1 File) and its subsequent performance in a multidimensional scaling plot (MDS) (Fig 2). AMOVAs for different grouping criteria (Table D in S1 File) and a Mantel test were also performed to determine the correlation between F_ST_ and geographic matrices. Finally, the number of shared haplotypes based on HVRI among the 28 stocks was calculated (Table E in S1 File).

### Evolutionary and demographic analysis

To analyze potential population size changes over time, we calculated a Bayesian skyline plot (BSP) using Bayesian Evolutionary Analysis Sampling Trees (BEAST 7.1) [55]. Estimations were carried out assuming the HKY+G evolutionary model, a long-normal relaxed molecular clock with a mean substitution rate of 3,02x10^−7^ mutation/site/year for the non-coding region [56]. For a detailed review of the choice of mutation rate, we used the research of Saint Pierre and colleagues [57].

The scaled effective population size was converted to Nef, assuming a generation time of 25 years. Importantly, an assumption about the mutation rate and the generation time will only affect the scale of the Bayesian skyline plot, but not its shape [58]. To assess the effect that sample size had on the construction of the skyline, different sequences were randomly selected to reach the total maximum value for each population (Figure A in S2 File.). For all the analyses, Markov Chain Monte Carlo (MCMC) samples were based on 60,000,000 iterations. Genealogies and model parameters were sampled every 1,000 iterations thereafter.

The use of the mtDNA control region for reconstructing demographic history by means of BSP is, in certain circumstances, very suitable. The BSP is fundamentally based on coalescence theory, which includes most of the properties that the BSP must meet. One of the most important is the selection and representativeness of the sample; the samples should ideally be obtained from individuals that have been randomly sampled from a panmictic population. In fact, the balanced sampling strategy whereby samples are distributed across several populations provides the best scheme for inferring demographic change over a typical time scale [59]. Sample selection should be carefully considered in relation to the population structure before BSP analyses are carried out. The samples presented here have been selected to fulfill this requirement.

Another essential aspect which is not usually taken into account when applying BSP is the possible existence of population sub-structure [60–62], since this directly influences the construction of the demographic model [63–66]. This work represents, in this sense, an advantage, since the samples come from a geographical region which provides valuable information about their genetic structure [33, 44, 45, 67].

Generally, however, increasing the amount of information in the alignment, either by increasing sequence length or by focusing on variable regions, will improve the precision of phylogenetic estimation of the genealogy.

In fact, like the mitogenomes, methodologically the control region is treated as if it were a single locus [68], providing enough information to reconstruct the demographic history of human populations [69]. In addition, the control region meets other important principles that make it suitable for BSP studies; it presents intraindividual variation and is not subject to selective pressure. On this last point, it should be noted that the effect of selection is to shift the distribution of mutations in the genealogy. For example, purifying selection leads to an excess of mutations near the tips of the genealogy [70,71].

One of the potential problems in using the control region is that certain drawbacks such as having a greater quantity of hotspots can alter the phylogenies and overestimate divergence times [72]. This problem can be resolved, however, especially when studying recent populations like the aboriginal Amerindians, by applying a mutation rate concordant with the evolutionary process, in this case mutation rates closer to those of pedigrees than phylogenies [57].

In addition, some other independent papers validate the use of the control region as a tool with which to reconstruct the demographic history of populations. One of these studies conducted on South American native populations provides results that largely coincide with our knowledge of the history of Amerindian populations [57]. In another study, the authors compared the results using complete mitogenomes and their control regions and found no differences between the BSPs [73]. Finally, a study comparing the BSPs generated from a few mitogenomes [74] with those created from a larger number of control regions from the same geographical area but from different populations [75], showed that both were practically the same.

A final point to note is that the control region has also been used to test correlations between demographic and paleoclimatic events [65,76], examining the factors driving past population dynamics [55,77,78], and tracing the transmission and spread of viruses [73,79].

**Fig 3:**
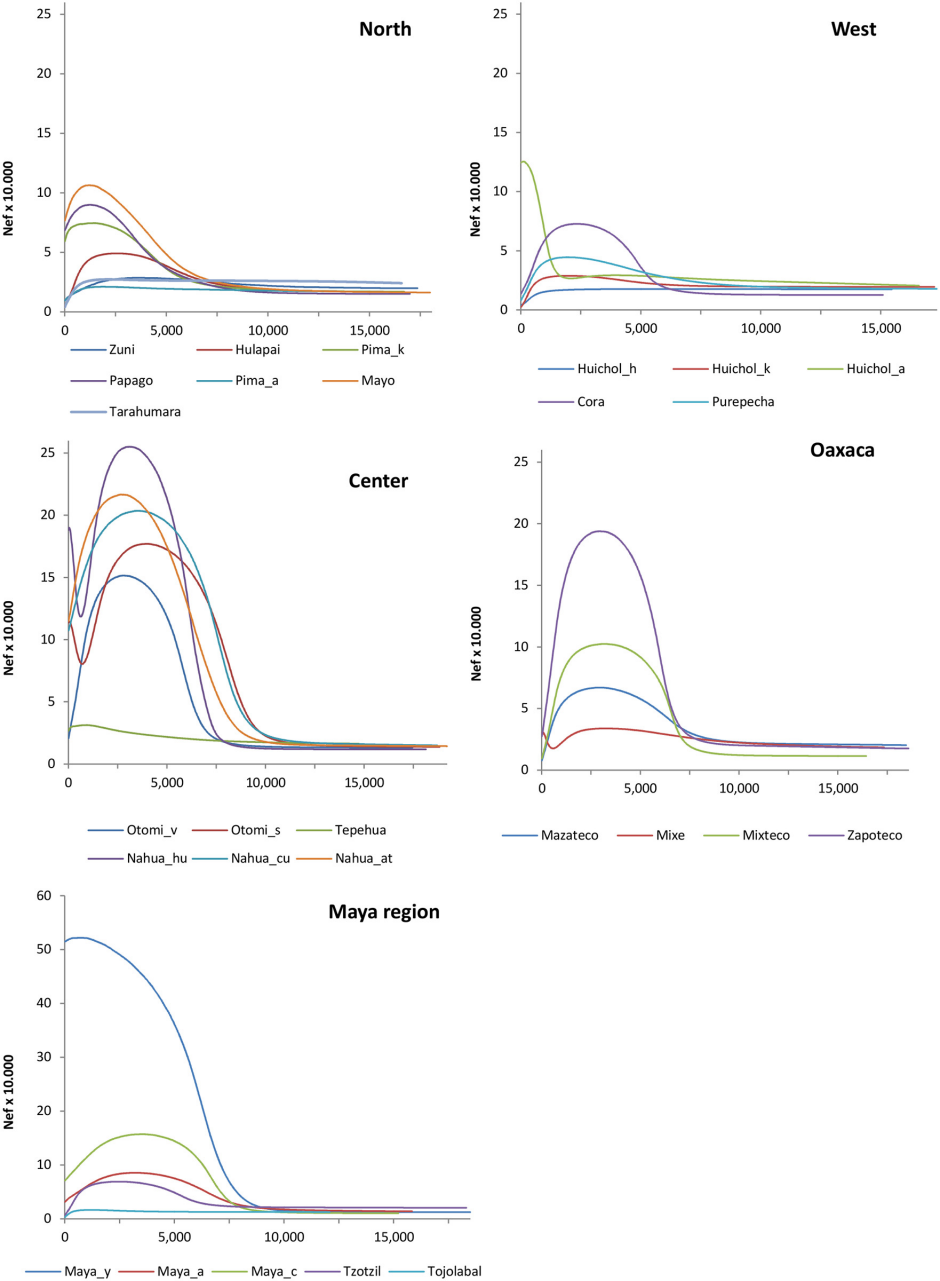
Skyline plot for the 28 populations grouped in the five cultural regions. North, West, Central, Oaxaca and Maya.

The skyline results, represented by the median values, are shown in graphs for cultural regions: North, West, Central, Oaxaca and Maya region (Fig 3). Different scales were used and Bayesian confidence intervals were omitted in order to compare population variations between indigenous groups. The skyline plot including the confidence intervals for each group in all cultural areas is represented in Figure B in S2 File.

**Fig 4:**
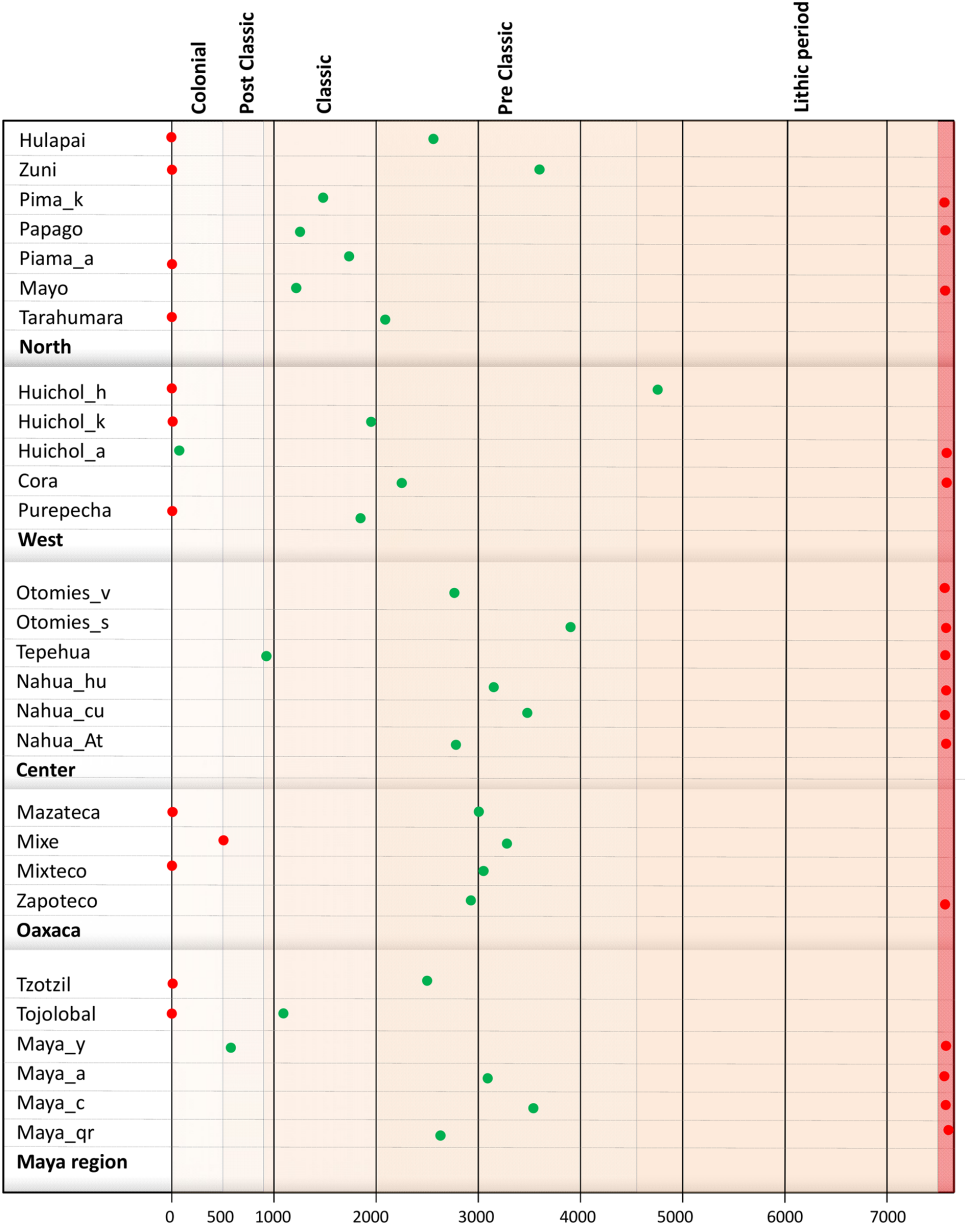
Time distribution (bottom) of the maximum (green points) and minimum Nef values (red points) by Mesoamerican periods (top) for populations clustered in cultural areas. The vertical red stripe indicates periods before 8,000 ybp.

Both maximum and minimum periods for Mesoamerica were represented in order to simplify the demographic history comparison between different groups (Fig 4). Maximum and minimum Nef values for each group can be found in Table F in S1 File. From the Nef generation estimation, demographic inter-generational growth rates (IGR) were calculated as follows: IGR = ((Nef _n_—Nef _n-1_) / Nef _n-1_)*100

Nef_n_ and Nef_n-1_ being the effective number of breeding females for a particular period and for the previous one, respectively. These calculations were performed for the 28 groups. The time evolution of the IGR for each of the 28 indigenous populations grouped by cultural area is shown in Figure C in S2 File.

**Fig 5:**
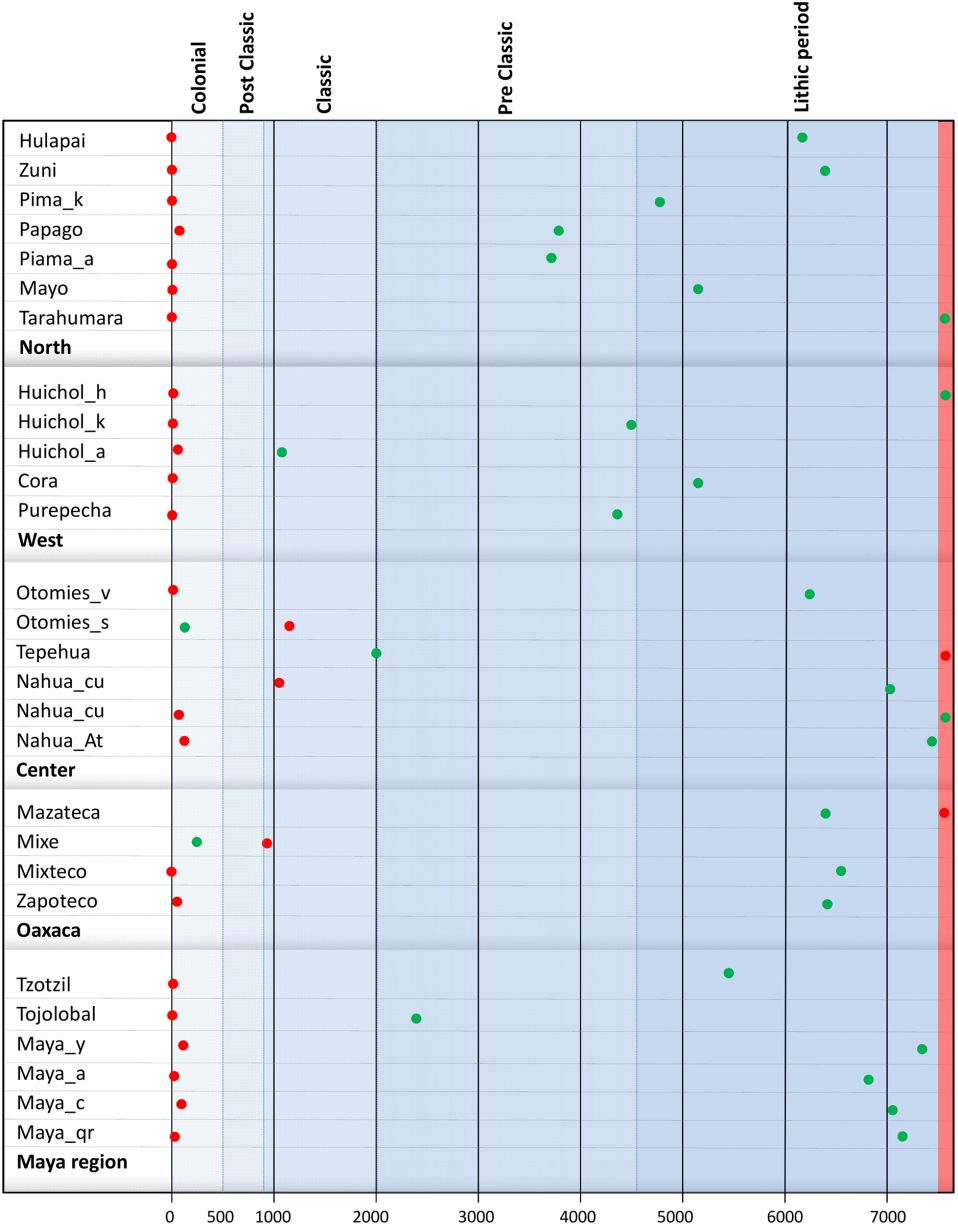
Time distribution (bottom) of the maximum (green points) and minimum IGR values (red points) by Mesoamerican periods (top) for populations clustered in cultural areas. The vertical red stripe indicates periods before 8,000 ybp.

The time distribution of the maximum and minimum IGR values (IGRmax and IGRmin) by Mesoamerican periods is presented in Fig 5. Detailed information on these data is presented in Table G in S1 File. From the point of view of the demographic development of the population and its interaction with the environment, it is important to determine when the values are reversed, i.e. at what point in the history of Mesoamerica the native groups gained or lost population. These inversion periods are represented in Figure D in S2 File. Note that the IGR values experiment intense fluctuations over time, especially in populations with small sizes. Therefore, the period in which the inversion occurred has been considered to be the one in which the rates have equal values, whether negative or positive.

## Results

We obtained the polymorphic sites in the mtDNA hypervariable regions I and II (HVRI and HVRII) detected in the 520 individuals from eight Native Mexican populations (Table A in S3 File). These complete mtDNA sequences will be available at EMPOP database (www.empop.org). In addition, 20 Mesoamerican populations were included in the global analyses. The geographic, cultural and linguistic information of the total population sample is shown in Fig 1 and Table 1.

The most common hg in the eight Mesoamerican populations was A2 (n = 257, 49.4%), followed by B2 (n = 126, 24.2%), C1 (n = 101, 19.4%) and D1 (n = 23, 4.4%), with significant intergroup variations (Table 2). Tojolabales present an increased B2 hg frequency (58.1%) and a total absence of hg C1. However, the most important deviation was observed in Huicholes, with a high proportion of C1 (47.1%) and absence of hg D1 (Χ^2^ = 45.518; p = 0.000). Complete sequencing of the control region allows us to determine the most important sub-haplogroups (subhgs), for example D4h3a and B4b1. D4h3a was found in twelve individuals (2.3%), eight in Tzotziles (9.2%) and four in Mazatecos (9.8%). Conversely, the subhg B4b1 was detected only in Mayas from the Yucatan (Table 2). Four African hgs were detected (data not shown), indicating small African maternal admixtures in Maya_y (2.44%), Tzotzil (1.14%) and Purepecha (2.98%). Due to the focus of anthropological studies on haplogroups of Native American lineage, the African hgs were omitted in all statistical analyses.

The highest genetic diversity rate (Ĥ) was detected in Mayan populations from the Yucatan Peninsula (Ĥ = 0.9974 ± 0.006), and the lowest in Tojolobal (Ĥ = 0.8911 ± 0.017) and Huichol_h (Ĥ = 0.7994 ± 0.026) (Table 3). However, using the nucleotide diversity index (π), this pattern changes, with the Native Mexican groups Mazateco (π = 0.01056 ± 0.0005) and Tzotzil (π = 0.01067 ± 0.0002) having the highest values. In contrast, Tojolabales present the lowest values for all genetic diversity indices. On the other hand, negative values were observed in the neutrality test (Tajima's D and Fu's FS) for the populations from the Yucatan Peninsula (Maya_qr, Maya_y and Maya_c) and Purepecha, indicating a relatively recent population growth. We must remember that for a population fall coming after a population expansion, the sign of the Tajima’s D value depends on the timing and degree of these events although it is difficult to place in time.

**Table 3:** Diversity parameters based on mtDNA control region sequences for the eight Native Mexican populations included in this study.

Population	n	k	S	Ĥ ± sd	π ± sd	θ	D	FS
**Mayan groups**								
**Quintana Roo**	74	40	68	0.9811±0.005	0.00913±0.0092	10.435	-0.8386	-7.10203
**Yucatan**	40	38	86	0.9974±0.006	0.00978±0.0084	11.326	-1.4943	-22.54797
**Campeche**	37	28	57	0.9819±0.001	0.00843±0.0006	9.674	-1.0887	-7.90736
**Tojolabales**	74	16	35	0.8911±0.017	0.00777±0.0004	8.954	0.8977	5.07651
**Tzotziles**	87	28	60	0.9513±0.008	0.01067±0.0002	12.171	0.0718	1.67121
**Other Mexican groups**								
**Mazatecos**	41	20	53	0.9585±0.013	0.01056±0.0005	12.412	0.0071	0.6201
**Purépechas**	65	23	52	0.9307±0.015	0.00661±0.0006	8.244	-0.7179	-0.70342
**Huicholes**	102	14	36	0.7994±0.026	0.00898±0.0002	11.213	2.0406	13.9224

n, sample size; k, number of different haplotypes; S, polymorphic sites; H, haplotype diversity; π, nucleotide diversity;

θ, mean number of pairwise differences between sequences. D, Tajima’s D; FS, Fu’s FS test (sd, standard deviation).

The only populations with no significant differences, expressed by corrected average pairwise differences, are those from the Yucatan Peninsula (Table B in S1 File). Genetic distances between the eight populations included in this study were represented in an MDS plot (Fig 2a). Two results in particular should be noted: i) the existence of a cluster formed by the Mayan populations from the Yucatan Peninsula (Maya_y, Maya_c and Maya_qr) and the other slightly remote Mayan populations, especially Tojolobals; and ii) the nearest population to the Mayan ones is the Mazatec, lending support to the idea of continuity between genetics and geography. The lowland western populations (Purepecha and Huichol_h) are not grouped together; in fact, Purepecha have more affinity with Mayans than with Huichol_h, who are far from the cluster (Fig 2a).

We performed some AMOVA tests for the eight Mesoamerican populations according to different clustering criteria (Table B in S1). The results were not significant when Mayan populations were compared with the rest (F_CT_ = 0.0125, *p* = 0.1368). However, significant results were found when the same cluster (Culture II) was divided into two Mayan population groups: those from the Yucatan Peninsula and the rest (F_CT_ = 0.0037, *p* = 0.0161). We also obtained significant results in the clusters 'Culture Area' (F_CT_ = 0.0062, *p* = 0.0148) and 'Language' (F_CT_ = 0.0480, *p* = 0.0098). A second set of analyses was then undertaken using these eight Mexican indigenous populations and a further 20 Native American groups. The latter came from the same geographical area and/or cultural background. The first step in this analysis was to calculate F_ST_ genetic distances (Table C in S1 File) and an MDS representation (Fig 2b). Most of the Nahua, Otomi, Maya and Oaxaca populations, the most representative Mesoamerican cultures, are grouped into a central cluster, indicating a large genetic homogeneity. Some groups lie outside this distribution, and although most of them are of northern origin (Hualapai, Zuni, Pimas_a, Huichol_h, Huichol_k, and Tarahumara), three belong to Mesoamerica (Tojolabal, Mixe and Purepecha). If we compare the position of the populations in the MDS with Ĥ values, the graph becomes particularly interesting. In fact, the Euclidean distance of each population from the coordinates of the center is negatively correlated with Ĥ (r = −0.985, *p* <0.0001), which is an indication that the populations at the periphery of the distribution are those with less diversity. Thus, the MDS reflects not only inter-population genetic relationships, but also the genetic diversity of the populations. This latter parameter is related to demographic history and shows the effects of genetic drift. Moreover, the Mantel test, contrasting the F_ST_ distance matrix and the geographical distances between the 28 Native American groups, showed a significant correlation (r = 0.207, *p* = 0.0003).

In order to estimate the relationship between populations, shared haplotypes from hypervariable region I (HVR I) were calculated (Table E in S1 File). The largest number of shared variants was detected between Otomí_v and Otomí_s (14); and between Nahua_hu and Otomí groups (14 and 13), as well as between Nahua_hu and Nahua_cu (10), Zapotec (9), and Mayan groups from the Yucatan Peninsula: Maya_qr (9), Maya_y (8), and Maya_c (8). The detected value between Pima_k and Papago (10), and between Nahua_at and Otomí groups (8 for both) is interesting. High values were also observed between the Yucatan Peninsula groups: Maya_c with Maya_y (9), and Maya_c with Maya_qr (8).

It is worth noting that different research teams have observed quite different values for the same indigenous group. For example, in the literature there is a pronounced range of observed values for the Huichol (represented by Huichol_k, Huichol_h and Huichol_a) and Pima groups (Pima_k and Pima_a) from western and northern regions, respectively. Conversely, population samples closer to the Mesoamerican core (from Center to Southeast, Mexico) such as Nahua (Nahua_cu, Nahua_at and Nahua_hu), Otomí (Otomi_v and Otomi_s) and Maya groups (Maya_a, Maya_qr, Maya_c, Maya_y, Tzozil) show more homogeneous results; Tojolabales were the only exception.

Two of the new set of AMOVA tests performed on the 28 Native American populations indicated significant differentiation between groups (Table D in S1 File): 'Pre-Hispanic area' (F_CT_ = 0.0858, *p* = 0.0000) and 'Cultural area' (F_CT_ = 0.0640, *p* = 0.0000), while 'Linguistic groups' displayed no-significant *p*-value (F_CT_ = 0.0088, *p* = 0.0840).

To get a more realistic picture of the complex demographic history of the indigenous populations, the BSP approach was applied (Fig 3). For this purpose, different random numbers of samples were selected in order to verify the effects on the skyline profile (S1 File). In this way, we verified that sample size does not influence the BSPs, validating that the studied samples are representative.

The observed demographic growth pattern over time agrees with that estimated by other authors and can be described in three stages [58,80,81]. A first stable phase is common to all the Native American populations. The second period is characterized by an increase in population, which is more or less pronounced depending on the indigenous group. This increase begins around 13,000 ybp with the fastest growth rate observed from 3,000 to 7,000 ybp. In the last phase, a demographic decline begins around 3,000 to 2,000 ybp depending on the cultural area and the indigenous group. However, this demographic growth model varies for the different study populations, in accordance with their different histories and subsistence strategies. It should be noted that each population has a different demographic history and cultural groupings have been made to address the results in a systematic manner. In fact, the boundaries and cultural parameters that define them have fluctuated depending on the conceptual evolution of disciplines and knowledge about Mesoamerica [19, 23]. The analysis of these results by cultural area showed the following findings:

**North**. This area includes seven indigenous groups (Fig 1, Table 1) showing a relatively low number of breeding females (Nef), in some cases with a minimum of 5,000 individuals (Fig 4, Table G in S1 File). The demographic model is similar in all locations: a peak is reached during the Preclassic and Classic periods (Fig 4), and the Nef minimum coincides with the Colonial period or the early Lithic one. IGRmax values of population increase present a logical time lapse with respect to the maximum Nef, and are detected in the Lithic and Preclassic periods (Fig 5). Despite some uniformity, Hualapai, Tarahumara and Zuni are rather different to the general demographic growth model. This uniqueness can be seen in the periods in which the inversion of the IGR trend occurs (Figure C and Figure D in S2 File, Table G in S1 File), in the Preclassic period for these populations and in the Classic one for the rest.

**West**. The demographic behavior of the five populations is very heterogeneous, particularly that of the Huichol_a and Cora. The former maintain positive IGRs until the present and reach relatively high population sizes (Nef = 125,000; 100 years ago) (Figs 4 and 5). This difference in the IGR values, compared with those of other populations of the same filiation (Huichol_h and Huichol_k), reflects the historical differences among Huichol communities. Furthermore, the Cora group maintains a constant IGR value, implying that it has had a higher population than the rest of the western indigenous groups.

**Center**. In this region, the majority of the groups have high population sizes, with the exception of the Tepehua who show a maximum Nef of 31,302 individuals, compared with the 255,025 individuals for Nahuas_hu (Table F in S1 File). Most of these populations have maximum IGR values during the lithic period (Fig 5). It is important to highlight the similar skylines of the Otomí_s and Nahua_hu, both with different linguistic affiliations but sharing the same geographical space (Fig 3).

**Oaxaca**. In this cultural region the demographic model is similar for all four populations, with maximum IGR values for the Lithic period and maximum population sizes in the Preclassic period during which the change is also detected (Figs 4 and 5). The exception to this model is the Mixe group, which shows minimum Nef values in the Classic period and maximum values in the Colonial one.

**Mayan**. A very different skyline is found for this region, with Tojolabales and Maya_y (Figs 4 and 5) showing the most divergent demographic history. The former have maintained discrete population sizes (maximum Nef of 16,406 in the late-Classic period), while the latter show constant growth rates over time, with a peak at the end of the Postclassic period (Nef = 522,057).

## Discussion

Despite the numerous studies that have been carried out regarding Mesoamerican indigenous groups, there are still doubts about their origins, genetic relationships and, above all, their demographic history. This paper analyses the phylogeographic and demographic history of eight Mesoamerican indigenous groups, five of Mayan descent and three from western Mexico.

The complete mitochondrial D-loop region of 520 Native Mexican individuals was sequenced, and different population indices were calculated, comparing them to 20 indigenous populations from neighboring regions. To our knowledge, this is the first time the Bayesian statistical method has been successfully applied to the D-loop region in order to reconstruct the demographic history of each of the Mexican indigenous groups (other authors have generally focused on macro-regions). In some cases the results were pooled by cultural region in order to facilitate a comparative analysis.

The 520 samples analyzed herein belong to one of the six Pan American hgs, confirming the common origin and close relationship between Native Mexican groups. This contrasts with the hypothesis formulated in other studies supporting the idea of a dual origin for Mesoamerican populations [17].

The small African admixture found in three Mexican indigenous groups (< 3%) is in agreement with previous results based on both mtDNA [67] and autosomal markers [82,83]. These results suggest that Post Columbian maternal gene flow toward Mexican indigenous communities preferentially has involved women of African and Native American origins due to social constraints.

Some authors have described a pattern in the mtDNA frequency distribution in North America that consists of a north-south gradient for hgA2, a south-north gradient for hgC1 and hgD1, and a lack of distribution for hgB2 [84, 85]. According to this proposal, the expected frequencies in Mesoamerica should be a high proportion of A2 and B2 and a lower presence of C1 and D1 [86]. In general our results agree with this model. However, when an analysis is made on a smaller geographical scale, this model is not observed. This result can be directly related to a phenomenon described in many human populations, that is, female mobility is mainly limited to small or medium distances [87].

Six hgs have been described in this paper (A2, B2, B4b1, C1, D1 and D4h3a) and, as expected, haplogroup X2a has not been detected. This result is not surprising, however, since its distribution is typically restricted to northeastern North America [88]. It is important to remember that the presence of this variant marks a possible continental route of expansion in the North American sub-continent.

D4h3 is a founder haplotype with an early presence in Native Americans [10, 12], and it is also considered a distinctive variant marking the Pacific human expansion into the new continent [7]. Although five of the eight populations studied herein are located on the Pacific coast, this variant has only been detected in two inland groups, Mazateco and Tzotzil (Fig 1, Table 2). The absence of D4h3 in other Native American populations could be explained by genetic drift effects, given the low frequency (2.3%) of this haplotype in Mesoamerican indigenous groups. Another possibility implies that after crossing the narrowest region of Mesoamerica, the Isthmus of Tehuantepec, the route of human expansion changed direction diagonally into the continent in order to avoid the coastal foothills of the current state of Chiapas. This hypothesis, supported by some researchers [89], seems to be the most plausible explanation since the oldest Mayan settlements were located in the southeast of the state of Chiapas [90]. This model of expansion would therefore imply an initial inward movement into the Mayan region, explaining the presence of D4h3 in Mazatec and Tzotzil populations, followed by a second expansion to the coast and the subsequent disappearance of the haplotype due to genetic drift effects.

Although the B4b1 subhg is a clade initially defined exclusively for southeast Asian populations [91], it has recently been detected in America in one Quechua sample (Chile). The most plausible hypothesis for its presence in the Yucatan Peninsula is that this clade traveled along Pacific trade routes established by the Europeans. Although in a previous study, Eurasian hgs were not observed in Mexican Mestizos from the Center to the Southeast [92], 1.4% of Asian and non-Amerindian genomic ancestry has been described in the Yucatan region [93].

Our results show a colonization model characterized by a certain basal homogeneity common to all populations, but with differences in terms of cultural sub-regions [89]. The process that explains this model would begin with the arrival to Mesoamerica of populations composed of a single genetic pool and distributed to new colonized regions. The significant correlation between geography and F_ST_ shows the importance that geography had in population differentiation. In this context, indigenous populations started differentiating within and between cultural areas, which favored the important linguistic and cultural diversity nowadays found in the region. The fixation indices calculated for the eight populations support this approach, yielding significant values mainly between populations of different cultural areas. Although no studies analyze this phenomenon in earlier periods, the studies carried out for the Classic and Postclassic period support the idea that population movements took place primarily within these cultural areas and were influenced by geography, trade and politics [39,40,94]. Trade, which stimulated migration and contact between human groups within and between cultural areas, is a factor that can largely explain the genetic structure of Mesoamerica expressed in the genetic homogeneity for the central area, Oaxaca and the Maya region.

The study of the influence of language also supports this idea; initially, at a micro-geographic level, language had an important role as a genetic barrier as suggested by the significant AMOVA results of the eight populations. However, the importance of this cultural component is diluted when the study region is expanded. These results are consistent with the hypothesis claiming that genetic differentiation in Native American groups occurred before the linguistic differentiation [45].

A second crucial event, which had important consequences in the population, was the adoption of agriculture. The development of maize domestication (*Zea mays*), and courgette (*Cucurbita pepo*), bean (*Phaseolus vulgaris*) and pepper (*Capsicum annuum*) crops led to a more sedentary lifestyle, an increase in social and urban complexity and the development of trade and migration routes. These trade-associated migrations were mainly performed within the same cultural areas and, on a second level, between areas [34,94]. This process is reflected in the AMOVA results, in which cultural areas significantly explain population stratification. This sub-structure can be seen in the MDS plots (Fig 2b), with a central cluster formed largely by the Oaxaca and Maya populations of the Center region.

Populations in the North and West regions, as well as exceptions from the Mayan region and Oaxaca, are distributed on the outskirts of this central cluster. The distribution of northern populations is consistent with the existence of a genetic barrier between the North region and Mesoamerica [67]. Differentiation between these two regions is justified by different survival strategies practiced by indigenous groups on both sides of this hypothetical barrier. The northern populations, hunter-gatherers until a few generations ago, remained small in size and underwent intense genetic drift effects. These characteristics, together with limited contact with other populations [95] as a consequence of geographical barriers, such as the mountains and canyons of the Sierra Madre, facilitated their low diversity and a high genetic differentiation relative to other Mesoamerican groups. In this sense, it is interesting to note the intermediate position of Mayo, Cora and Tarahumara between both regions (Fig 2b).

Populations that are not from the North but are located on the periphery of the main group, probably have low genetic diversity due to other circumstances. Northern populations have almost no hgs shared with other groups, suggesting a process of differentiation and past isolation. The Huichol_h, Huichol_k, Mixe and Tojolabales show low diversity but share hgs with other groups from their area and from other cultural regions. The behavior of these groups suggests that the isolation process occurred in more recent times. Another possible explanation for the shared hgs of these populations could be recent gene flow, as suggested by some studies based on GWAS [33]. Other studies corroborate the idea of the importance of trade and politics, especially in the Postclassic period, in shaping the current genetic structure of Mesoamerica [34]. These studies mostly suggest contact between Mayan populations and the central-eastern region of Mesoamerica, as well as a migration route between the Center and the West. These migrations probably of little importance quantitatively, however, since otherwise, as well as having shared hgs indicating a common ancestry, increased levels of diversity should also be detected.

Within the context of population genetics, demographic history studies are essential in order to obtain a specific reconstruction of the history of indigenous groups. In addition to estimating Nef per generation, IGRs were calculated to reconstruct population fluctuations in detail. Population reconstruction using Bayesian methods interprets demographic growth as the adaptive reflection of the populations; colonization of new ecosystems with resources and without competition and/or improvements in how they are exploited [58,81,82].

In general, most of the indigenous cultural groups from south and southeast areas, such as Oaxaca and the Mayas, show high values of Nef in the Preclassical period, with some exceptions such as Maya_y, Tojolobales, and Tepehuas. During that time, Mesoamerican societies adopted an agriculture lifestyle as a survival strategy and thereby no longer required a nomadic existence to search for food and other resources, resulting in an increase in the social complexity and population density. The latter is interesting because the population growth detected in the present work has been corroborated by independent studies [96]. These peaks tend to move towards the Classic period in western groups and especially in those from the North region. The maximum population in the West coincides with the development of the Teuchitlán or Tumbas de Tiro tradition (2,400–1,800 ybp), whereas results for the North are related to population expansion from Mesoamerica toward the north [97]. In all cases, the maximum demographic values do not coincide with the period of greatest splendor of Mesoamerican cultures, but rather are found in preceding periods.

On the other hand, Nef minimum values are distributed in either the Lithic period, possibly representing the founder effect, or in recent periods, reflecting the population decline suffered in many Mexican indigenous groups that has continued until today. In order to measure in real time the adaptive level of populations, the IGR has to be estimated, with Nef the consequence of the accumulation of positive or negative values of these rates over a specific period. Although it is virtually impossible to conduct a systematic analysis of the behavior of IGRs by indigenous group, the detailed IGR evolution is shown (Figure C in S2 File). The interpretation is focused on IGRmax and IGRmin as well as periods in which there was a change in trend.

IGRmax values are found in general during the Lithic period, and the positive trend continues until the Preclassic. After this period, the values decrease and are negative, reaching the current IGRmin. This model is valid for groups from cultural regions of central, southern and southeastern Mesoamerica, whereas in the West and North regions maximum Nef values and fluctuations over time are detected. In the West region, IGRmax is reached at the beginning of the Preclassic and the inversion occurs at the end of this period and the early Classic. In the North, IGRmax values show a wide range of temporal variation from the Lithic to the Preclassic, with the inversion at the end of the Preclassic or early Classic (Figure D in S2 File and Table G in S1 File).

The information provided by the IGRs generates some interesting conclusions. The first is that Mesoamerican populations quickly adapted to their environment in the early stages of colonization. Moreover, this growth was more or less stable throughout their history until periods prior to the splendor of the Mesoamerican cultures; after that, they began a slow and steady decline in population. This early population growth coincides with the beginning of agriculture and a sedentary lifestyle [24]. In fact, the timing difference detected between the West and the rest of Mesoamerica could be related to the onset of agriculture dated between 3,650–3,250 ybp [98] (Capacha culture) in the West [99] and 8,240 ybp, 6,208 ybp and 5,090 ybp for Oaxaca, the Center and the Maya region, respectively [100].

Another interesting question is the growth model for the northern groups, which clearly reflects their hunter-gatherer strategy: old IGRmax, dating back to the lithic period, and relatively low values of IGR that maintain discrete population increases.

One of the advantages of having different populations representing the same native group is that one can approach the study of intragroup internal structure. The analysis of shared hgs reveals that populations of the same group tend to share hgs, an indication of consistency. However, their distribution in the MDS and demographic behavior suggests two models. The first is a fairly homogeneous model, which is represented by the inherited stocks of large cultures of central Mesoamerica: Mayan, Aztec and the Otomi, including the western Purepecha group. The genetic and demographic similarities detected among Otomi and Nahua from the Sierra Madre Oriental are an interesting result. This coincidence, even for culturally different groups, reflects a constant gene flow between both groups favored by geographical proximity and corroborated by the high number of shared haplogroups.

A second model is represented by northern groups, such as Huicholes and Pimas, who have a heterogeneous demographic behavior. That is, the development of large cultures involves genetic homogeneity; conversely, less cohesive cultures show variations in their demographic patterns pointing to periods of isolation and independence among communities presumably from the same indigenous group. Huichol are a particularly interesting group. Ethnographic and ethnohistorical studies indicate the possibility that Huichols derive from various groups who settled in the Sierra Madre Occidental [101,102]. The detected heterogeneity for this group could be a reflection of this story, in fact, mtDNA [67] and Y chromosome studies [103] confirm the dual origin of this group.

A final point to note is that, although the arrival of Europeans had a major impact on the demography of indigenous population (the negative IGRmax values are detected after the contact), the demographic decline began in Mesoamerica a few hundred years before. This result can be contrasted with paleo-climatic studies conducted in both Mesoamerica and the Mayan region. In Mesoamerica it has been suggested that during the middle Holocene weather conditions were relatively stable, but that a dry period began about 5,800 ybp [104]. These dates coincide with the periods estimated using Bayesian statistics in which the IGRs begin to decline and eventually turn negative. Regarding the Mayas region, it is estimated that the collapse of this culture occurred in the years 1,110 to 1,200 [96], which practically coincides with the values detected for the demographic decline of the Yucatan Mayas (625 ybp).

In summary, our study–applying for the first time Bayesian skyline methodology to mitochondrial D-loop sequences–highlights the demographic changes that took place over time and in different geographical areas of Mesoamerica. These were the result of the complex interrelationship between geography, subsistence strategies, social structure and culture.

## Supporting Information

S1 File **Table A. Genetic differentiation among the Mesoamerican populations studied herein.** Corrected pairwise differences average (below diagonal) and p-values (above diagonal), among the eight Mexican Native populations based on the control region of mtDNA sequences.
**Table B. Population structure among the eight Mesoamerican populations studied herein.** AMOVA based on historic, geographic, cultural and linguistic criteria for the eight indigenous populations studied herein.
**Table C. Genetic differentiation among 28 Native American populations**. F_ST_ values between the 28 Native American populations based on the control region of mtDNA sequences.
**Table D. Population structure among Native American populations based on different criteria**. AMOVA based on historic, geographic, cultural and linguistic and criteria among 28 Native American populations included in this study.
**Table E. Genetic relationships based on shared haplotypes between Native American populations.** Number of shared haplotypes between the 28 Native American populations based on HVRI data
**Table F. Estimates of Nef values for each of the Native American groups studied herein**. Female effective population size (Nef) and corresponding maximum and minimum estimated for the studied Native American populations based on mitochondrial control region data.
**Table G. Demographic and temporary parameters estimated in the Native American groups studied herein**. Temporary distribution of female effective population size (Nef), intergenerational growth rates (IGR) with maximum and minimum values, and period in which the IGR trend inversion occurred (major demographic changes) estimated in the Native American groups studied herein based on mitochondrial control region data.</SI_Caption>
(PDF)

S2 File **Figure A. Bayesian skyline plot based on random samples from the 28 studied indigenous populations.** A different number of samples were randomly selected to determine that sample size has no effect on the demographic profile.
**Figure B. The 28 studied indigenous populations grouped by cultural areas.** The Nef median value is represented in the Bayesian skyline plot including confidence intervals.
**Figure C. IGR values temporary evolution for each indigenous group grouped by cultural areas.** The Y-axis represents IGR percentage value and X-axis time in ybp.
**Figure D. Temporary distribution (bottom) according to Mesoamerican periods (top) in which the trend inversion occurred.** The vertical red stripe indicates periods previous to 8,000 ybp.</SI_Caption>
(PDF)

S3 File Table A. Polymorphic sites in mtDNA hypervariable regions I and II (HVRI y HVRII) defining Native American haplogroups detected in eight Mexican indigenous populations. Absolute frequencies observed in each Mexican population studied herein are presented.
(XLSX)

## References

[1] O’Rourke DH, Raff JA. The human genetic history of the Americas: the final frontier. Curr Biol. 2010;20: R202–7. 10.1016/j.cub.2009.11.051 20178768

[2] Goebel T, Waters MR, O'Rourke DH. The late Pleistocene dispersal of modern humans in the Americas. Science. 2009;319(5869): 1497–1502. 10.1126/science.1153569 18339930

[3] Achilli A, Perego UA, Lancioni H, Olivieri A, Gandini F, Kashani B et al Reconciling migration models to the Americas with the variation of North American native mitogenomes. Proc Natl Acad Sci USA. 2013;110(35): 14308–14313. 10.1073/pnas.1306290110 23940335 PMC3761611

[4] Reich D, Patterson N, Campbell D, Tandon A, Mazieres S, Ray N et al Reconstructing Native American population history. Nature. 2012;488(7411): 370–374. 10.1038/nature11258 22801491 PMC3615710

[5] Tamm ET, Kivisild T, Reidla M, Metspalu M, Smith DG, Mulligan C et al Beringian standstill and spread of Native American founders. PLoS ONE. 2007;2: e829 17786201 10.1371/journal.pone.0000829 PMC1952074

[6] Bodner M, Perego UA, Huber G, Fend L, Rock AW, Zimmermann B et al Rapid coastal spread of first Americans: novel insights from South America’s Southern cone mitochondrial genomes. Genome Res. 2012;22: 811–820. 10.1101/gr.131722.111 22333566 PMC3337427

[7] Perego UA, Achilli A, Angerhofer N, Accetturo M, Pala M, Olivieri A et al Distinctive Paleo-Indian migration routes from Beringia marked by two rare mtDNA haplogroups. Curr Biol. 2009;19: 1–8. 10.1016/j.cub.2008.11.058 19135370

[8] Dillehay TD. Probing deeper into first American studies. Proc Natl Acad Sci USA. 2009;106(4): 971–978. 10.1073/pnas.0808424106 19164556 PMC2633551

[9] Bandelt HJ, Herrnstadt C, Yao YG, Kong QP, Kivisild T, Rengo C et al Identification of Native American founder mtDNAs through the analysis of complete mtDNA sequences: Some caveats. Ann Hum Genet. 2003;67: 512–524. 14641239 10.1046/j.1469-1809.2003.00049.x

[10] Rasmussen M, Anzick SL, Waters MR, Skoglund P, DeGiorgio M, Stafford TW et al The genome of a Late Pleistocene human from a Clovis burial site in western Montana. Nature. 2014;506: 225–229. 10.1038/nature13025 24522598 PMC4878442

[11] Raff JA, Bolnick DA. Palaeogenomics: Genetic roots of the first Americans. Nature. 2014;506: 162–163. 10.1038/506162a 24522593

[12] Kemp BM, Malhi RS, McDonough J, Bolnick DA, Eshleman JA, Rickards O et al Genetic analysis of early holocene skeletal remains from Alaska and its implications for the settlement of the Americas. Am J Phys Anthropol. 2007;132: 605–621. 17243155 10.1002/ajpa.20543

[13] Chatters JC, Kennett DJ, Asmerom Y, Kemp BM, Polyak V, Blank AN et al Late Pleistocene human skeleton and mtDNA link Paleoamericans and modern Native Americans. Science. 2014;344(6185): 750–754. 10.1126/science.1252619 24833392

[14] Malhi RS, Cybulski JS, Tito RY, Johnson J, Harry H, Dan C. Brief communication: mitochondrial haplotype C4c confirmed as a founding genome in the Americas. Am J Phys Anthropol. 2010;141: 494–497. 10.1002/ajpa.21238 20027611

[15] Perego UA, Angerhofer N, Pala M, Olivieri A, Lancioni H, Kashani BH et al The initial peopling of the Americas: a growing number of founding mitochondrial genomes from Beringia. Genome Res. 2010;20: 1174–1179. 10.1101/gr.109231.110 20587512 PMC2928495

[16] Kumar S, Bellis C, Zlojutro M, Melton P E, Blangero J, Curran J. Large scale mitochondrial sequencing in Mexican Americans suggests a reappraisal of Native American origins. BMC Evol Biol. 2012;11: 293. 10.1186/1471-2148-11-293 PMC3217880 21978175

[17] Mizuno F, Gojobori J, Wang L, Onishi K, Sugiyama S, Granados J et al Complete mitogenome analysis of indigenous populations in Mexico: its relevance for the origin of Mesoamericans. J Hum Genet. 2014;59(7): 359–367. 10.1038/jhg.2014.35 24804703

[18] Kirchhoff P. Mesoamerica: its geographic limits ethnic composition and cultural characteristics. Illinois: The Free Press Publishers; 1952.

[19] Carmack RM, Gossen GH, Gasco J. The legacy of Mesoamérica: history and culture of a Native American civilization. Nueva Jersey: Prentice Hall; 1996.

[20] Creamer W. Mesoamerica as a Concept: An archeological view from Central America. Lat Am Res Rev. 1987;22(1): 35–62.

[21] Blanto RE, Kowalewski SA, Feinman GM, Finsten LM. A comparison of change in three regions. Cambridge: Cambridge University Press; 1981.

[22] Cambell LC, Kaufman T, Smith-Stark T. Meso-america as a Linguistic Area. Language. 1986;62(3):530–570.

[23] Coe MD. Mexico: from the Olmecs to the Aztecs. New York: Thames and Hudson; 1996

[24] López-Austin A, López Luján L. Mexico’s Indigenous Past. Oklahoma: University of Oklahoma Press; 2001.

[25] FAMSI-LACMA. Foundation for the Advancement of Mesoamerica Studies-Los Angeles County Museum of Art. Available: http://www.famsi.org

[26] Trigger BG. Archaeology and the image of the American Indian. Am Antiqu. 1980;45(4): 662–676.

[27] Kroeber A. Cultural and natural areas of native North America. Berkeley: University of California Press; 1947.

[28] Adams REW. Prehistoric mesoamerica. Oklahoma: University of Oklahoma; 1996.

[29] Sanders WT. Mesoamerica: the evolution of a civilization. Nueva York: Random house; 1968.

[30] Aguilar-Moreno M. A Handbook to life in the Aztec world. Oxford: Oxford University Press; 2004.

[31] Kirchhoff P. Mesoamérica: sus límites geográficos composición étnica y caracteres culturales. Acta Americana. 1943;1: 92–107.

[32] Duverger C. El primer mestizaje, la clave para entender el pasado mesoamericano. México: Ed Taurus; 2007.

[33] Moreno-Estrada A, Gignoux CR, Fernández-López JC, Zakharia F, Sikora M, Contreras AV et al The genetics of Mexico recapitulates Native American substructure and affects biomedical traits. Science. 2014;344(6189): 1280–1285. 10.1126/science.1251688 24926019 PMC4156478

[34] Ragsdale CS, Edgar HJH. Cultural interaction and biological distance in PostClassic period Mexico. Am J Phys Anthropol. 2015 10.1002/ajpa.22701 25599818

[35] Solanes Carraro MC, Vela E. Atlas del México prehispánico. Arqueología Mexicana, 2005.

[36] Manzanilla L, López Luján L. Atlas Histórico de Mesoamérica. Mexico: Larousse; 1989

[37] Smith ME, Berdan FF. PostClassic Mesoamerican World system. Curr Anthropol. 2000;41:283 10702149

[38] Smith ME. The Aztec empire and the Mesoamerican world system In: Alcock SE, D’Altroy DN, Morrison KD, Sinopoli CM editors. Empires: comparative perspective from archaeology and history. Cambrigde: Cambridge University Press; 2001 pp. 128–154

[39] Melgar Tisoc ER. Una Relectura del comercio de la Turquesa: entre yacimientos, talleres y consumidores Caminos y Mercados de Mexico. México: Universidad Autonoma de Mexico; 2010.

[40] McGuire RH. Mesoamerica and the Southwest/Northwest In: Nichols, Pool CA, editors. The oxford handbook of Mesoamerican archaeology. Oxford: Oxford University Press; 2012 pp. 513–524.

[41] Wauchope R. Handbook of Middle American Indians. Austin: University of Texas Press; 1964.

[42] West CR, Augelli JP. Middle America: Its Lands and Peoples. New York: Prentice Hall; 1989.

[43] Weaver MP. The Aztecs, Maya, and Their Predecessors: Archaeology of Mesoamerica. San Diego: Academic Press; 1993.

[44] Kemp BM, González-Oliver A, Malhi RS, Monroe C, Schroeder KB, McDonough J et al Evaluating the farming/language dispersal hypothesis with genetic variation exhibited by populations in the Southwest and Mesoamerica. Proc Natl Acad Sci USA. 2010;107: 6759–6764. 10.1073/pnas.0905753107 20351276 PMC2872417

[45] Sandoval K, Buentello-Malo L, Peñaloza-Espinosa R, Avelino H, Salas A, Calafell F et al Linguistic and maternal genetic diversity are not correlated in Native Mexicans. Hum Genet. 2009;126: 521–531. 10.1007/s00439-009-0693-y 19495796 PMC2762527

[46] Henn BM, Cavalli-Sforza LL, Feldman MW. The great human expansion. Proc Natl Acad Sci USA. 2012;109(44): 17758–17764 10.1073/pnas.1212380109 23077256 PMC3497766

[47] Gronau I, Hubisz MJ, Gulko B, Danko CG, Siepel A. Bayesian inference of ancient human demography from individual genome sequences. Nat Genet. 2011;43(10): 1031–1034. 10.1038/ng.937 21926973 PMC3245873

[48] Tillmar AO, Coble MD, Wallerström T, Homlund G. Homogeneity in mitochondrial DNA control region sequences in Swedish subpopulations. Int J Legal Med. 2010; 124: 91–98. 10.1007/s00414-009-0354-7 19590886

[49] Irwin JA, Saunier JL, Strouss KM, Sturk KA, Diegoli TM, Just RS et al Development and expansion of high-quality control region databases to improve forensic mtDNA evidence interpretation. Forensic Sci Int Genet. 2007;1: 154–157. 10.1016/j.fsigen.2007.01.019 19083747

[50] Edson SM, Ross JP, Coble MD, Parsons TJ, Barritt SM. Naming the dead—Confronting the realities of rapid identification of degraded skeletal remains. Forensic Sci Rev. 2004;16: 64–89. 26256813

[51] Brandstätter A, Peterson CT, Irwin JA, Mpoke S, Koech DK, Parson W et al Mitochondrial DNA control region sequences from Nairobi (Kenya): inferring phylogenetic parameters for the establishment of a forensic database. Int J Legal Med. 2004;118: 294–306. 15248073 10.1007/s00414-004-0466-z

[52] Brandstätter A, Niederstätter H, Pavlic M, Grubwieser P, Parson W. Generating population data for the EMPOP database—an overview of the mtDNA sequencing and data evaluation processes considering 273 Austrian control region sequences as example. Forensic Sci Int. 2007;166: 164–175. 16829006 10.1016/j.forsciint.2006.05.006

[53] Librado P, Rozas J. DNAsp v5: A software for comprehensive analysis of DNA polymorphism data. Bioinformatics. 2009; 25:1451–1452. 10.1093/bioinformatics/btp187 19346325

[54] Excoffier L, Lischer HEL. Arlequin suite ver. 3.5: A new series of programs to perform population genetics analyses under Linux and Windows. Mol Ecol Resour. 2010;10: 564–567. 10.1111/j.1755-0998.2010.02847.x 21565059

[55] Atkinson QD, Gray RD, Drummond AJ. mtDNA variation predicts population size in humans and reveals a major southern Asian chapter in human prehistory. Mol Biol Evol. 2008;25: 468–474. 18093996 10.1093/molbev/msm277

[56] Endicott P, Ho SYW. A Bayesian Evaluation of Human Mitochondrial Substitution Rates. Am J Hum Genet. 2008;82: 895–902. 10.1016/j.ajhg.2008.01.019 18371929 PMC2427281

[57] de Saint Pierre M, Gandini F, Perego UA, Bodner M, Gómez-Carballa A, Corach D et al Arrival of Paleo-Indians to the southern cone of South America: new clues from mitogenomes. PLoS One. 2012;7(12):e51311 10.1371/journal.pone.0051311 23240014 PMC3519775

[58] O’Fallon BD, Fehren-Schmitz L. Native Americans experienced a strong population bottleneck coincident with European contact. Proc Natl Acad Sci USA. 2011;108: 20444–20448. 10.1073/pnas.1112563108 22143784 PMC3251087

[59] Heller R, Chikhi L, Siegismund HR. The confounding effect of population structure on Bayesian Skyline Plot inferences of demographic history. PLoS ONE. 2013;8(5): e62992 10.1371/journal.pone.0062992 23667558 PMC3646956

[60] Miller HC, Moore JA, Allendorf FW, Daugherty CH. The evolutionary rate of tuatara revisited. Trends Genet. 2009;25:13–15. 10.1016/j.tig.2008.09.007 18976831

[61] Subramanian S. Temporal trails of natural selection in human mitogenomes. Mol Biol Evol. 2009;26:715–717. 10.1093/molbev/msp005 19150805

[62] Subramanian S, Hay JM, Mohandesan E, Millar CD, Lambert DM. Molecular and morphological evolution in tuatara are decoupled. Trends Genet. 2009;25:16–18.

[63] Strimmer K, Pybus OG. Exploring the demographic history of DNA sequences using the generalized skyline plot. Mol Biol Evol. 2001;18:2298–2305. 11719579 10.1093/oxfordjournals.molbev.a003776

[64] Pannell JR. Coalescence in a metapopulation with recurrent local extinction and recolonization. Evolution. 2003;57: 949–961. 12836814 10.1111/j.0014-3820.2003.tb00307.x

[65] Drummond AJ, Rambaut A, Shapiro B, Pybus OG. Bayesian coalescent inference of past population dynamics from molecular sequences. Mol Biol Evolution. 2005; 22:1185–1192. 10.1093/molbev/msi103 15703244

[66] Navascués M, Emerson BC. Elevated substitution rate estimates from ancient DNA: model violation and bias of Bayesian methods. Mol Ecol. 2009;18:4390–4397. 10.1111/j.1365-294X.2009.04333.x 19735451

[67] Gorostiza A, Acunha-Alonzo V, Regalado-Liu L, Tirado S, Granados J, Samano D et al Reconstructing the History of Mesoamerican Populations through the Study of the Mitochondrial DNA Control Region. PLoS ONE. 2012;7(9): e44666 10.1371/journal.pone.0044666 23028577 PMC3446984

[68] Ho SYW, Shapiro B. Skyline-plot methods for estimating demographic history from nucleotide sequences. Mol Ecol Resour. 2011;11: 423–434. 10.1111/j.1755-0998.2011.02988.x 21481200

[69] Heled J, Drummond AJ. Bayesian inference of population size history from multiple loci. BMC Evol Biol. 2008;8:289 10.1186/1471-2148-8-289 18947398 PMC2636790

[70] Fu YX, Li WH. Statistical test of neutrality of mutations. Genetics. 1993;133:693–709. 8454210 10.1093/genetics/133.3.693 PMC1205353

[71] Williamson S, Orive ME. The genealogy of a sequence subject to purifying selection at multiple sites. Mol Biol Evol. 2002;19: 1376–1384. 12140250 10.1093/oxfordjournals.molbev.a004199

[72] Soares P, Ermini L, Thomson N, Mormina M, Rito T, Röhl A et al Correcting for purifying selection: an improved human mitochondrial molecular clock. Am J Hum Genet. 2009;84: 740–759. 10.1016/j.ajhg.2009.05.001 19500773 PMC2694979

[73] Kitchen A, Miyamoto MM, Mulligan CJ. A Three-Stage colonizacion model for the peopling of the Americas. PLoS ONE. 2008;3(2): e1596 10.1371/journal.pone.0001596 18270583 PMC2223069

[74] Gunnarsdóttir ED, Li M, Bauchet M, Finstermeier K, Stoneking M. High-throughput sequencing of complete human mtDNA genomes from the Philippines. Genome Res. 2011;21(1): 1–11. 10.1101/gr.107615.110 21147912 PMC3012916

[75] Guillot EG. Climate change influenced female population sizes through time across the Indonesian Archipelago. Hum Biol. 2013;85(1–3): 135–152. 24297223 10.3378/027.085.0306

[76] Campos PF, Willerslev E, Sher A, Orlando L, Axelsson E, Tikhonov A et al Ancient DNA analyses exclude humans as the driving force behind late Pleistocene musk ox (*Ovibos moschatus*) population dynamics. Proc Natl Acad Scien USA. 2010;107: 5675–5680. 10.1073/pnas.0907189107 PMC2851807 20212118

[77] Finlay EK, Gaillard C, Vahidi SM, Mirhoseini SZ, Jianlin H, Qi XB et al Bayesian inference of population expansions in domestic bovines. Biol Lett. 2007;3: 449–452. 17535790 10.1098/rsbl.2007.0146 PMC2111054

[78] Stiller M, Baryshnikov G, Bocherens H, Grandal d'Anglade A, Hilpert B, Münzel SC et al Withering away –25 000 years of genetic decline preceded cave bear extinction. Mol Biol Evol. 2010;27: 975–978. 10.1093/molbev/msq083 20335279

[79] Magiorkinis G, Magiorkinis E, Paraskevis D, Ho SY, Shapido B, Pybus OG et al The global spread of hepatitis C virus 1a and 1b: a phylodynamic and phylogeographic analysis. PLoS Medicine. 2009;6: e1000198 10.1371/journal.pmed.1000198 20041120 PMC2795363

[80] Fagundes NJ, Kanitz R, Bonatto SL. A reevaluation of the Native American mtDNA genome diversity and its bearing on the models of early colonization of Beringia. PLoS One. 2008;3(9): e3157 10.1371/journal.pone.0003157 18797501 PMC2527677

[81] Fagundes NJ, Kanitz R, Eckert R, Valls AC, Bogo MR, Salzano FM et al Mitochondrial population genomics supports a single pre-Clovis origin with a coastal route for the peopling of the Americas. Am J Hum Genet. 2008;82(3): 583–592. 10.1016/j.ajhg.2007.11.013 18313026 PMC2427228

[82] Pickrell JK, Pritchard JK. Inference of population splits and mixtures from Genome-Wide allele frequency data. PLoS Genetics. 2012;8: e1002967 10.1371/journal.pgen.1002967 23166502 PMC3499260

[83] Rosenberg NA, Pritchard JK, Weber JL, Cann HM, Kidd KK, Zhivotovsky LA et al Genetic structure of human populations. Science. 2002;298: 2381–2385. 12493913 10.1126/science.1078311

[84] Malhi RS, Schultz BA, Smith DG. Distribution of mitochondrial DNA lineages among Native American tribes of Northeastern North America. Hum Biol. 2001;73(1): 17–55. 11332644 10.1353/hub.2001.0008

[85] Malhi RS, Eshleman JA, Greenberg JA, Weiss DA, Schultz Shook BA, Kaestle FA et al The structure of diversity within New World mitochondrial DNA haplogroups: implications for the prehistory of North America. Am J Hum Genet. 2002;70(4): 905–919. 11845406 10.1086/339690 PMC379119

[86] Shields GF, Schmiechen AM, Frazier BL, Redd A, Voevoda MI, Reed JK et al mtDNA sequences suggest a recent evolutionary divergence for Beringian and northern North American populations. Am J Hum Genet. 1993;53(3): 549–562. 8352271 PMC1682422

[87] Lippold S, Xu H, Ko A, Li M, Renaud G, Butthof A et al Human paternal and maternal demographic histories: insights from high-resolution Y chromosome and mtDNA sequences. Investig Genet. 2014;5:13 10.1186/2041-2223-5-13 25254093 PMC4174254

[88] Reidla M, Kivisild T, Metspalu E, Kaldman K, Tambets K, Tolk HV et al Origin and diffusion of mtDNA haplogroup X. Am J Hum Genet. 2003;73: 1178–1190. 14574647 10.1086/379380 PMC1180497

[89] Wang S, Lewis CM, Jakobsson M, Ramachandran S, Ray N, Bediya G et al Genetic variation and population structure in Native Americans. PLoS Genet. 2007;3(11): e185 18039031 10.1371/journal.pgen.0030185 PMC2082466

[90] Clark J, Hansen RD, Pérez Suárez T. La zona maya en el preclásico In Manzanilla, López Luján L., editors. Historia antigua de México Vol I. El México antiguo, sus áreas culturales, los orígenes y el horizonte Preclásico. México: INAH-UNAM; 2000 pp. 437–510

[91] Derenko M, Malyarchuk B, Denisova G, Perkova M, Rogalla U, Grzybowski T et al Complete Mitochondrial DNA Analysis of Eastern Eurasian Haplogroups Rarely Found in Populations of Northern Asia and Eastern Europe. PLoS ONE. 2011;7(2): e32179 10.1371/journal.pone.0032179 PMC3283723 22363811

[92] Martínez-Cortés G, Haro-Guerrero JO, Salazar-Flores J, Rubi-Castellanos R, Velarde-Félix JS, Muñoz-Valle JF et al Maternal admixture and population structure in Mexican-Mestizos based on mtSNPs. Am J Phys Anthrop. 2013;151(4):526–37. 10.1002/ajpa.22293 23754474

[93] Silva-Zolezzi I, Hidalgo-Miranda A, Estrada-Gil J, Fernández-Lopez JC, Uribe-Figueroa L, Contreras A et al Analysis of genomic diversity in Mexican Mestizo populations to develop genomic medicine in Mexico. Proc Natl Acad Sci USA. 2009;106(21): 8611–8616. 10.1073/pnas.0903045106 19433783 PMC2680428

[94] Smith ME, Berdan FF. The PostClassic Mesoamerican world. 1st ed Salt Lake City: University of Utah Press; 2003.

[95] González-José R, Martínez-Abadías N, González-Martín A, Bautista-Martínez J, Gómez-Valdés J, Quinto M et al Detection of a population replacement at the Classic-PostClassic transition in Mexico. Proc Biol Sci. 2007;274(1610): 681–688. 17254992 10.1098/rspb.2006.0151 PMC2197204

[96] Kennett DJ, Breitenbach SF, Aquino VV, Asmerom Y, Awe J, Baldini J et al Development and disintegration of Maya political systems in response to climate change. Science. 2012;338(6108): 788–791. 10.1126/science.1226299 23139330

[97] Townsend R. Ancient West Mexico: Art and Archaeology of the Unknown Past. New York: Thames & Hudson; 1998.

[98] Evans ST, Webster DL. Archaeology of Ancient Mexico and Central America. New York: Garland Published; 2001.

[99] Kelly IT. Ceramic sequence in Colima: Capacha, and early phase. Tucson: Anthropological papers of the University of Arizona; 1980.

[100] Blake M. Dating the initial spread of Zea mays In Staller JE, Tykot RH, editors Histories of maize. San Diego: Academic Press; 2006 pp. 55–68.

[101] Schaefer SB, Stacy B, Furst PT. People of the Peyote Huichol Indian History, Religion, and Survival. Albuquerque: University of New Mexico Press; 1996.

[102] Neurath J. Huicholes Pueblos Indígenas del México Contemporáneo. Mexico: CONADEPI; 2003.

[103] Páez-Riberos LA, Muñoz-Valle JF, Figuera LE, Nuño-Arana I, Sandoval-Ramírez L, González-Martín A et al Y-linked haplotypes in Amerindian chromosomes from Mexican populations: genetic evidence to the dual origin of the Huichol tribe. Leg Med. 2006;8(4): 220–225. 10.1016/j.legalmed.2006.02.003 16797211

[104] Voorhies B, Metcalfe SE. Culture and climate in Mesoamerica during the Middle Holocene In: Anderson D, Maasch KA, Sandweiss DH, editors. Climate Change and Cultural Dynamics. San Diego: A Global Perspective on Mid-Holocene Transitions; 2007 pp. 157–187.

