# Supplementary material for: Demographic History of Indigenous Populations in Mesoamerica Based on mtDNA Sequence Data
Source: PLoS One. 2015 Aug 20;10(8):e0131791. doi: 10.1371/journal.pone.0131791 (PMC4546282; doi:10.1371/journal.pone.0131791)
Supplement: S1 File — Table A. Genetic differentiation among the Mesoamerican populations studied herein. Corrected pairwise differences average (below diagonal) and p-values (above diagonal), among the eight Mexican Native populations based on the control region of mtDNA sequences. Table B. Population structure among the eight Mesoamerican populations studied herein. AMOVA based on historic, geographic, cultural and linguistic criteria for the eight indigenous populations studied herein. Table C. Genetic differentiation among 28 Native American populations. FST values between the 28 Native American populations based on the control region of mtDNA sequences. Table D. Population structure among Native American populations based on different criteria. AMOVA based on historic, geographic, cultural and linguistic and criteria among 28 Native American populations included in this study. Table E. Genetic relationships based on shared haplotypes between Native American populations. Number of shared haplotypes between the 28 Native American populations based on HVRI data Table F. Estimates of Nef values for each of the Native American groups studied herein. Female effective population size (Nef) and corresponding maximum and minimum estimated for the studied Native American populations based on mitochondrial control region data. Table G. Demographic and temporary parameters estimated in the Native American groups studied herein. Temporary distribution of female effective population size (Nef), intergenerational growth rates (IGR) with maximum and minimum values, and period in which the IGR trend inversion occurred (major demographic changes) estimated in the Native American groups studied herein based on mitochondrial control region data. (PDF) [file pone.0131791.s001.pdf]

Table A. Corrected pairwise differences average (below diagonal) and p-values (above diagonal), among the eight Native Mexican populations based on control region of mtDNA sequences (*significance 0.05).

	Maya_y	Maya_c	Maya_qr	Tojolabal	Tzotzil	Mazateca	Purepecha	Huichol
**Maya_y**	---	0.40274	0.76149	0.0000*	0.0009*	0.0351	0.0009*	0.0000*
**Maya_c**	-0.02003	---	0.18866	0.0000*	0.0000*	0.0009*	0.0019*	0.0000*
**Maya_qr**	-0.11020	0.09675	---	0.0000*	0.0000*	0.0244	0.0000*	0.0000*
**Tojolabal**	**3.01232**	**3.98419**	**2.57100**	---	0.0000*	0.0000*	0.0000*	0.0000*
**Tzotzil**	**0.88280**	**1.31075**	**0.86272**	**1.79463**	---	0.0019*	0.0000*	0.0000*
**Mazateca**	0.60602	**1.13206**	0.46218	**1.21540**	**0.92908**	---	0.0000*	0.0000*
**Purepecha**	**0.77662**	**0.67696**	**0.96305**	**5.83687**	**2.83165**	**2.00823**	---	0.0000*
**Huichol**	**2.40699**	**2.95443**	**2.47122**	**3.09375**	**1.39987**	**2.75520**	**4.60707**	---

Table B. AMOVAs based on different classification criteria (*significance level 0.05) for the eight studied populations.

Grouping criteria	Groups	Populations		Variance	Fixation indices	P
Culture I	Mayan population	maya_qr, maya_c, maya_y, tzotzil, tojolobal	Within populations	92.18	F_{ST} = 0.0782	0.0000*
	No Mayan population	purepecha, huichol, mazateco	Among populations within groups	6.57	F_{SC} = 0.0665	0.0000*
			Among groups	1.25	F_{CT} = 0.0125	0.1368
Culture II	Yucatan peninsula	maya_qr, maya_c, maya_y	Within populations	92.61	F_{ST} = 0.0739	0.0000*
	Mayan population	tzotzil, tojolobal	Among populations within groups	7.02	F_{SC} = 0.0704	0.0000*
	No Mayan population	purepecha, hichol, mazateco	Among groups	0.37	F_{CT} = 0.0037	0.0161*
Culture Area	Yucatan peninsula	maya_qr, maya_c, maya_y	Within populations	92.59	F_{ST} = 0.0741	0.0000*
	Rest of Mayas of the south	tzotzil, tojolobal	Among populations within groups	6.79	F_{SC} = 0.0683	0.0000*
	Occident	purepecha, hichol,	Among groups	0.62	F_{CT} = 0.0062	0.0148*
	Oaxaca	mazateco				
Language	Maya	maya_qr, maya_c, maya_y, tzotzil, tojolobal	Within populations	91.24	F_{ST} = 0.0876	0.0000*
	Oto-Mangue	mazateco	Among populations within groups	3.96	F_{SC} = 0.0416	0.0000*
	Yuto-nahua	huichol	Among groups	4.80	F_{CT} = 0.0480	0.0098*
	Purépecha	purepecha				
Culture and language	Yucatan Peninsula	maya_qr, maya_c, maya_y	Within populations	92.25	F_{ST} = 0.0774	0.0000*
	Others mayan populations	tzotzil, tojolobal	Among populations within groups	3.73	F_{SC} = 0.0388	0.0000*
	Oto-mangue	mazateco	Among groups	4.02	F_{CT} = 0.0402	0.0948
	Yuto-Nahua	huichol				
	Tarascan	purepecha				

Table C. F_{ST} values for the 28 populations included in this study.

	Zuni	Hualapai	Pima_k	Papago	Pima_a	Tarahumara	Mayo	Cora	Huichol_k	Huichol_a	Huichol_h	Purpecha	Otomi_v	Otomi_s	Tepehua	Nahua_at	Nahua_cu	Nahua_hu	Mazateco	Mixe	Mixteco	Zapoteco	Tojolobal	Tzotzil	Maya_y	Maya_c	Maya_qr	Maya_a
**Zuni**	0.000																											
**Hualapai**	0.150	0.000																										
**Pima_k**	0.108	0.056	0.000																									
**Papago**	0.126	0.064	0.010	0.000																								
**Pima_a**	0.162	0.096	0.067	0.074	0.000																							
**Tarahumara**	0.138	0.088	0.056	0.066	0.087	0.000																						
**Mayo**	0.109	0.066	0.037	0.042	0.076	0.056	0.000																					
**Cora**	0.130	0.068	0.038	0.042	0.078	0.066	0.046	0.000																				
**Huichol_k**	0.104	0.101	0.069	0.077	0.111	0.097	0.072	0.067	0.000																			
**Huichol_a**	0.128	0.062	0.032	0.036	0.072	0.060	0.037	0.028	0.030	0.000																		
**Huichol_h**	0.210	0.147	0.115	0.123	0.156	0.145	0.124	0.100	0.084	0.058	0.000																	
**Purpecha**	0.141	0.078	0.047	0.055	0.087	0.079	0.058	0.049	0.092	0.053	0.137	0.000																
**Otomi_v**	0.120	0.059	0.031	0.037	0.065	0.060	0.039	0.040	0.054	0.016	0.102	0.050	0.000															
**Otomi_s**	0.118	0.057	0.030	0.035	0.067	0.057	0.036	0.039	0.071	0.032	0.115	0.049	0.023	0.000														
**Tepehua**	0.139	0.075	0.047	0.053	0.084	0.076	0.055	0.055	0.064	0.026	0.116	0.066	0.030	0.045	0.000													
**Nahua_at**	0.119	0.055	0.023	0.030	0.063	0.056	0.034	0.030	0.065	0.026	0.109	0.045	0.024	0.023	0.041	0.000												
**Nahua_cu**	0.118	0.051	0.021	0.027	0.061	0.051	0.029	0.032	0.066	0.025	0.115	0.042	0.022	0.020	0.039	0.017	0.000											
**Nahua_hu**	0.108	0.050	0.024	0.029	0.058	0.048	0.028	0.032	0.060	0.021	0.100	0.043	0.017	0.020	0.035	0.019	0.013	0.000										
**Mazateco**	0.136	0.070	0.041	0.047	0.080	0.062	0.041	0.046	0.085	0.043	0.130	0.062	0.042	0.039	0.058	0.038	0.030	0.031	0.000									
**Mixe**	0.143	0.079	0.050	0.056	0.088	0.080	0.058	0.053	0.093	0.054	0.139	0.070	0.051	0.049	0.067	0.047	0.042	0.043	0.062	0.000								
**Mixteco**	0.125	0.062	0.034	0.040	0.067	0.056	0.042	0.044	0.074	0.034	0.121	0.054	0.035	0.034	0.051	0.030	0.026	0.028	0.046	0.054	0.000							
**Zapoteco**	0.114	0.053	0.024	0.029	0.063	0.054	0.032	0.026	0.060	0.020	0.102	0.045	0.025	0.024	0.042	0.017	0.016	0.017	0.037	0.043	0.026	0.000						
**Tojolobal**	0.160	0.097	0.068	0.073	0.108	0.099	0.078	0.058	0.100	0.062	0.133	0.089	0.070	0.068	0.084	0.060	0.063	0.058	0.082	0.089	0.074	0.056	0.000					
**Tzotzil**	0.129	0.067	0.039	0.045	0.077	0.068	0.047	0.048	0.078	0.040	0.122	0.059	0.038	0.038	0.053	0.035	0.031	0.033	0.048	0.056	0.044	0.034	0.070	0.000				
**Maya_y**	0.109	0.045	0.014	0.021	0.055	0.046	0.024	0.026	0.060	0.019	0.108	0.035	0.017	0.014	0.034	0.010	0.007	0.008	0.027	0.034	0.019	0.011	0.056	0.025	0.000			
**Maya_c**	0.119	0.054	0.020	0.029	0.064	0.052	0.030	0.034	0.067	0.027	0.113	0.043	0.026	0.024	0.042	0.020	0.016	0.018	0.034	0.045	0.028	0.020	0.066	0.034	0.008	0.000		
**Maya_qr**	0.114	0.053	0.025	0.030	0.063	0.054	0.032	0.035	0.067	0.028	0.113	0.045	0.025	0.024	0.041	0.021	0.017	0.016	0.033	0.044	0.028	0.019	0.059	0.021	0.006	0.014	0.000	
**Maya_a**	0.128	0.065	0.034	0.043	0.077	0.068	0.047	0.049	0.082	0.042	0.129	0.057	0.038	0.036	0.053	0.033	0.030	0.021	0.051	0.059	0.043	0.029	0.073	0.048	0.018	0.029	0.026	0.000

Table D. AMOVAs based on different classification criteria (*significance level 0.05) the twenty eight populations included in this study.

Grouping criteria	Groups	Populations		Variance	Fixation indices	P
Prehispanic area	Mesoamerica	cora, huichol_k, huichol_a, huichol_h, purepecha, otomi_v, otomi_s, tepehuas, nahua_at, nahua_cu, nahua_hu, mazateca, mixe, mixteco, zapoteco, tojolobal, tzotzil, maya_a, maya_qr, maya_y, maya_c, tarahumara, mayo	Within populations	76.36	F_{ST} = 0.2364	0.0000*
	Oasisamerica	hualapai, zuni, pima_k, pima_a, papago,	Among populations within groups	15.06	F_{SC} = 0.1647	0.0000*
			Among groups	8.59	F_{CT} = 0.0858	0.0000*
Geographic and Cultural area	Maya	tojolobal, tzotzil, maya_a, maya_qr, maya_c, maya_y	Within populations	80.27	F_{ST} = 0.1972	0.0000*
	Oaxaca	mazateco, mixe, mixteco, zapoteco	Among populations within groups	13.32	F_{SC} = 0.1423	0.0000*
	Centro	nahua_at, nahua_cu, nahua_hu, otomi_v, otomi_s	Among groups	6.40	F_{CT} = 0.0640	0.0000*
	Gulf	tepehua				
	Occident	cora, huichol_k, huichol_a, huichol_h, pureprecha				
	North	mayo, tarahumara				
	South USA	hualapai, zuni, pima_k, pima_a, papago				
Languages	Aztec-tanoan	zuni	Within populations	94.19	F_{ST} = 0.0580	0.0000*
	YCochimí-Yuman	hualapai	Among populations within groups	4.93	F_{SC} = 0.0497	0.0000*
	Uto-aztecan	pima_k, pima_a, papago, tarahumara, mayo, cora, huichol_k, huichol_h, huichol_a, nahua_at, nahua_cu, nahua_hu	Among groups	0.88	F_{CT} = 0.0088	0.0840
	Tarascan	purepecha				
	Otomanguean	otomi_s, otomi_v, mazateco, zapoteco, mixteco				
	Totonacan, Tepehua	tepehua				
	Mixe-Zoquean	mixe				
	Mayan	maya_qr, maya_a, maya_y, maya_c, tojolabal, tzotzil				

Table E. Number of shared haplotypes between the twenty eight Native American populations based on HVRI data.

		North							Occident					Center						Oaxaca				Mayas					
		Zuni	Hualapai	Pima_k	Papago	Pima_a	Tarahumaras	Mayo	Cora	Huichol_k	Huichol_a	Huichol_h	Purépecha	Otomí_v	Otomí_s	Tepehua	Nahua_at	Nahua_cu	Nahua_hu	Mazateco	Mixe	Mixteco	Zapoteco	Tojolobal	Tzotzil	Maya_y	Maya_c	Maya_qr	Maya_a
North	Zunis																												
	Hulapai	3																											
	Pima_k	2	3																										
	Papago	1	1	10																									
	Pima_a	1	1	1	1																								
	Tarahumara	2	2	1	1	2																							
	Mayo	2	3	2	2	3	4																						
Occident	Cora	1	1	2	3	1	2	3																					
	Huichol_k	2	2	2	1	1	1	2	3																				
	Huichol_a	0	0	1	2	1	1	2	3	6																			
	Huichol_h	2	3	2	2	2	3	4	4	7	6																		
	Purépecha	1	2	1	0	1	1	2	1	1	0	2																	
Center	Otomí_v	2	4	4	2	3	1	3	3	4	3	5	5																
	Otomí_s	1	2	2	2	2	2	4	4	4	4	6	3	14															
	Tepehua	1	2	0	1	2	2	3	4	3	3	5	3	6	7														
	Nahua_at	1	2	2	1	3	1	2	2	4	2	4	4	8	8	5													
	Nahua_cu	1	1	2	1	1	1	2	4	4	3	4	2	6	7	3	5												
	Nahuas_hu	1	3	2	2	2	2	5	4	4	4	6	7	13	14	8	7	10											
Oaxaca	Mazateco	1	1	1	1	0	1	1	4	1	1	2	2	3	3	3	3	3	5										
	Mixe	1	2	2	1	2	1	3	2	3	1	3	4	6	5	3	6	4	6	5									
	Mixteco	1	3	1	1	2	1	3	3	4	3	5	3	6	6	4	5	4	7	2	5								
	Zapoteco	1	3	1	2	2	2	3	3	4	4	5	2	7	7	4	6	3	9	3	4	7							
Mayas	Tojolobal	1	2	1	1	1	0	2	2	2	1	2	2	4	4	3	4	2	5	1	3	5	5						
	Tzotil	1	2	2	1	1	0	2	3	3	2	3	2	5	6	3	4	3	7	1	2	4	6	3					
	Maya_y	2	2	1	1	2	2	2	3	2	1	3	4	7	6	3	4	4	8	3	4	4	4	3	3				
	Maya_c	1	2	1	2	2	0	4	3	3	3	5	3	4	7	4	3	4	8	2	4	5	5	5	3	9			
	Maya_qr	1	1	0	1	0	1	2	3	2	4	3	3	5	7	5	3	3	9	2	2	5	5	5	5	4	8		
	Maya_a	1	2	1	1	1	2	2	2	1	1	3	2	3	4	3	2	1	5	2	2	2	4	1	3	4	6	6	

Table F. Estimates for maximum and minimum Nef values for each of the indigenous groups studied.

		**Maximum**	**Minimum**
**North**	Hulapai	28.634	9.487
	Zuni	49.146	4.863
	Pima_k	74.564	16.877
	Papago	89.956	15.075
	Pima_a	21.115	9.433
	Mayo	106.322	16.274
	Tarahumara	27.301	4.226
**Occident**	Huichol_h	17.621	3.989
	Huichol_k	28.949	2.302
	Huichol_a	125.412	20.556
	Cora	72.703	12.585
	Purepecha	44.511	8.188
**Center**	Otomies_v	151.472	13.260
	Otomies_s	177.007	13.818
	Tepehua	31.302	16.395
	Nahua_hu	255.025	11.838
	Nahua_cu	203.549	15.009
	Nahua_at	216.576	14.362
**Oaxaca**	Mazateco	66.954	7.749
	Mixe	33.796	17.596
	Mixteco	102.445	9470
	Zapoteco	193.865	17.496
**Maya region**	Maya_y	522.057	12.188
	Maya_a	85.325	13.804
	Maya_c	157.116	10.201
	Maya_qr	170.224	11.184
	Tzolzil	69.017	6.341
	Tojolobal	16.409	2.765

Table G. Temporary distribution for Nef and IGR maximum and minimum values and period in which the IRG trend inverted. Nef, effective number female; IGR, intergenerational rate of population increase. Data in YBP.

			Nef		IGR	
		Tendency	Maximum	Minimum	Maximum	Minimum
North	Hulapai	2,500-2,475	2,550	Current	6,025	Current
	Zuni	3,400-3,375	3,700	Current	6,350	Current
	Pima_k	1,250-1,225	1,425	16,700	4,775	Current
	Papago	1,175-1,150	1,275	15,075	3,725	75
	Pima_a	1,850-1,825	1,875	Current	3,675	Current
	Mayo	1,200-1,175	1,225	17,975	5,100	Current
	Tarahumara	2,100-2,075	2,100	Current	15,175	Current
Occident	Huichol_h	3,950-3,925	4,725	Current	13,450	Current
	Huichol_k	1,9,75-1,950	1,975	Current	4,450	Current
	Huichol_a	100-75	100	16,575	1,100	25
	Cora	2,300-2,275	2,300	14,475	5,200	Current
	Purepecha	1,925-1,900	1,875	Current	4,300	Current
Center	Otomies_v	2,750-2,725	2,750	16,250	6,225	Current
	Otomies_s	0-25	3,975	18,825	275	1,225
	Tepehua	875-850	925	16,395	2,000	Current
	Nahua_hu	3,025-3,000	3,075	18,125	7,050	1,050
	Nahua_cu	3,575-3,550	3,475	18,700	8,050	75
	Nahua_at	2,650-2,625	2,775	14,362	7,725	150
Oaxaca	Mazateco	2,925-2,900	3,000	Current	6,325	25
	Mixe	3,000-2,975	3,250	577	250	900
	Mixteco	3,150-3125	3,150	Current	6,575	Current
	Zapoteco	2,925-2,900	2,925	18,575	6,375	25
Maya region	Maya_y	425-400	625	18,475	7,525	150
	Maya_a	3,125-3,100	3,125	15,825	6,825	25
	Maya_c	3,400-3,375	3,600	15,200	7,075	100
	Maya_qr	2,550-2,500	2,650	17,050	7,200	25
	Tzolzil	2,450-2,425	2,450	Current	5,550	Current
	Tojolobal	1,175-1,150	1,175	Current	2,350	Current
