# Supplementary material for: Demographic History of Indigenous Populations in Mesoamerica Based on mtDNA Sequence Data
Source: PLoS One. 2015 Aug 20;10(8):e0131791. doi: 10.1371/journal.pone.0131791 (PMC4546282; doi:10.1371/journal.pone.0131791)
Supplement: S2 File — Figure A. Bayesian skyline plot based on random samples from the 28 studied indigenous populations. A different number of samples were randomly selected to determine that sample size has no effect on the demographic profile. Figure B. The 28 studied indigenous populations grouped by cultural areas. The Nef median value is represented in the Bayesian skyline plot including confidence intervals. Figure C. IGR values temporary evolution for each indigenous group grouped by cultural areas. The Y-axis represents IGR percentage value and X-axis time in ybp. Figure D. Temporary distribution (bottom) according to Mesoamerican periods (top) in which the trend inversion occurred. The vertical red stripe indicates periods previous to 8,000 ybp. (PDF) [file pone.0131791.s002.pdf]

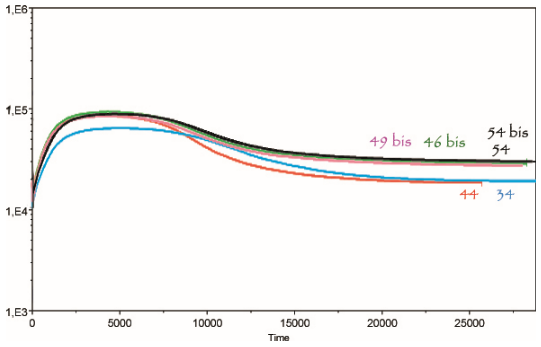

Hualapai

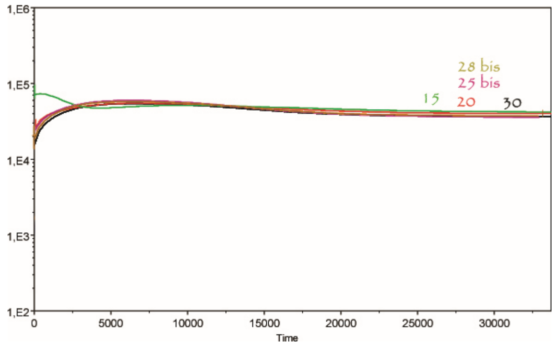

Zuni

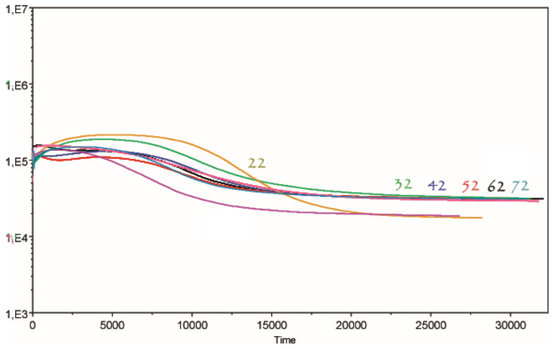

Pima_k

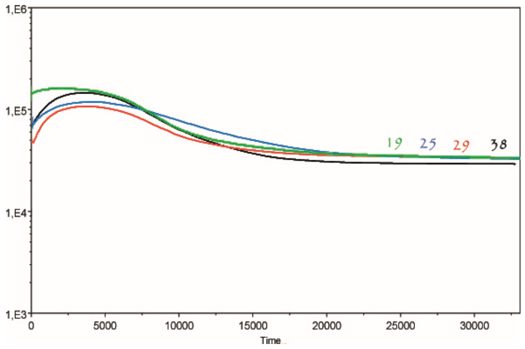

Papagos

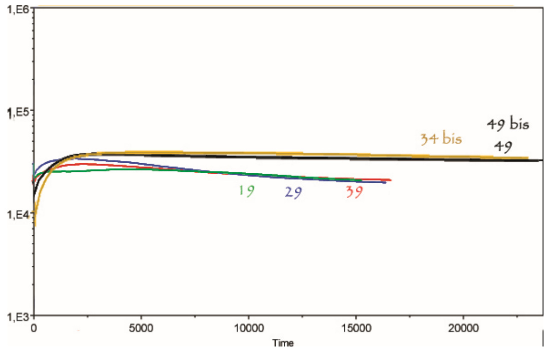

Pima_a

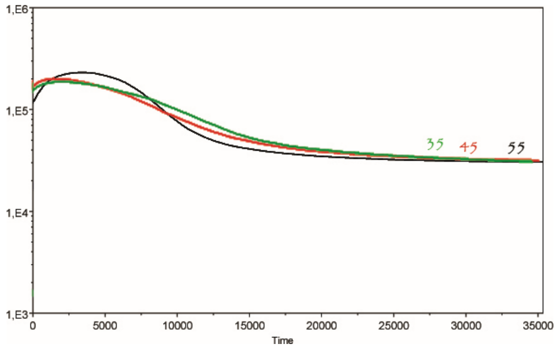

Mayo

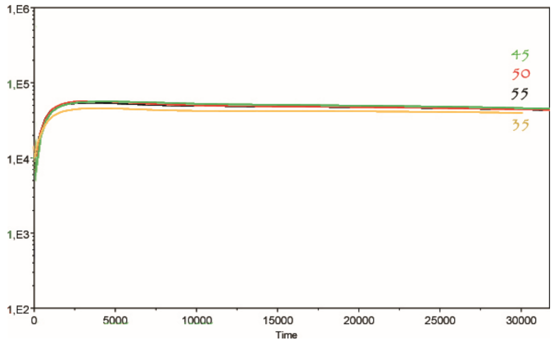

Tarahumara

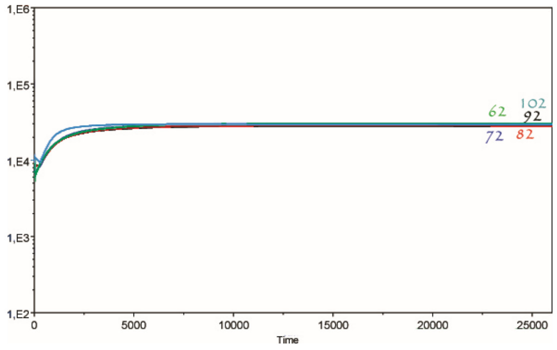

Huichol_h

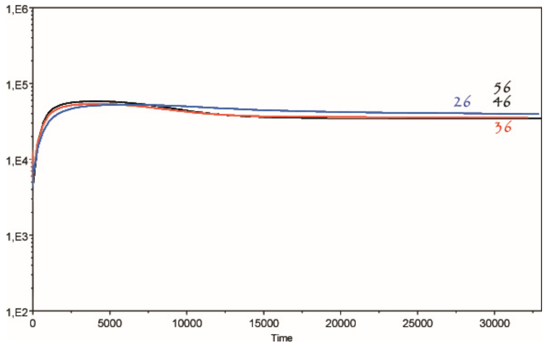

Huichol_k

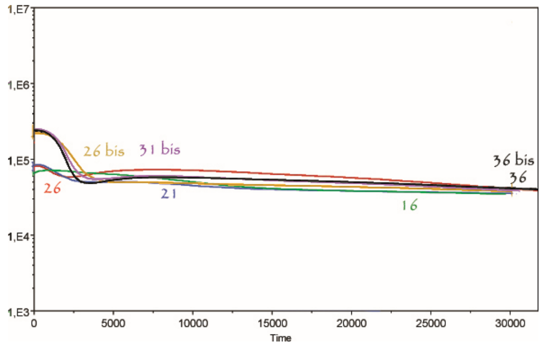

Huichol_a

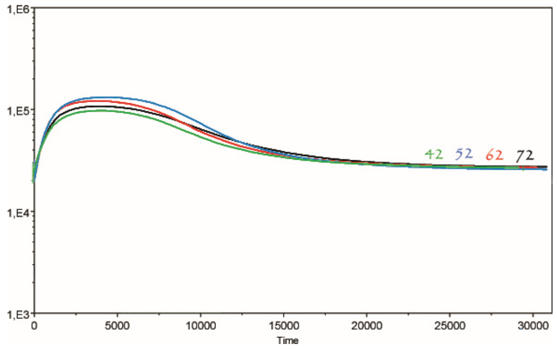

Cora

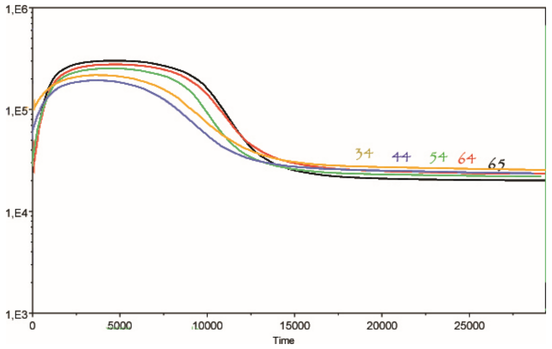

Purepecha

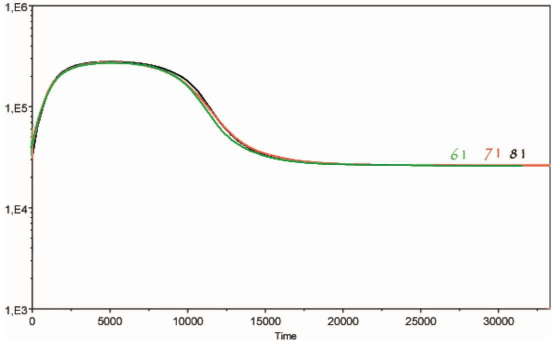

Otomí_v

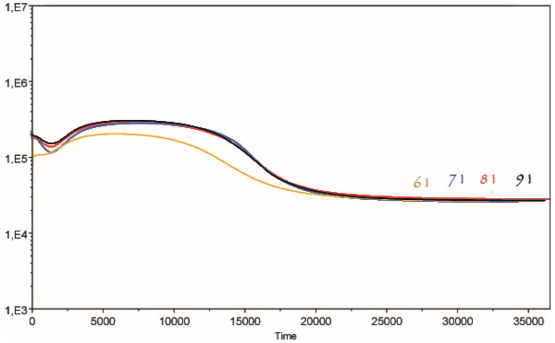

Otomí_s

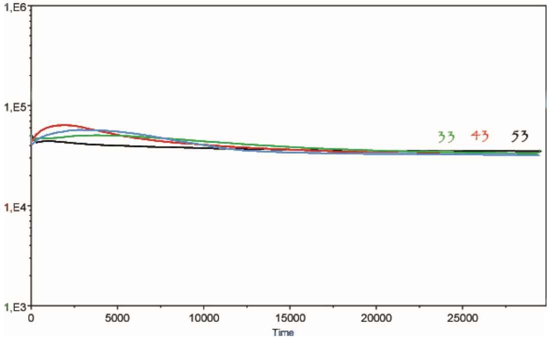

Tepehua

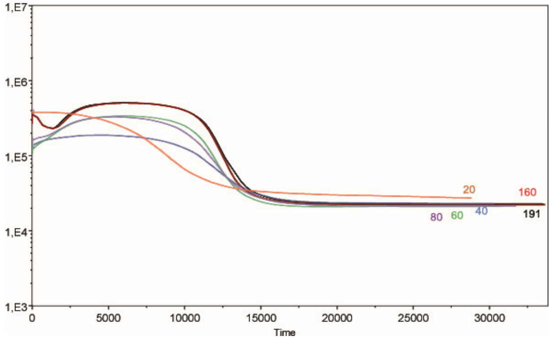

Nahua_hu

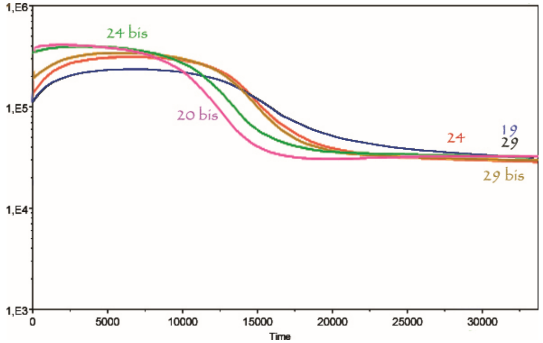

Nahu_cu

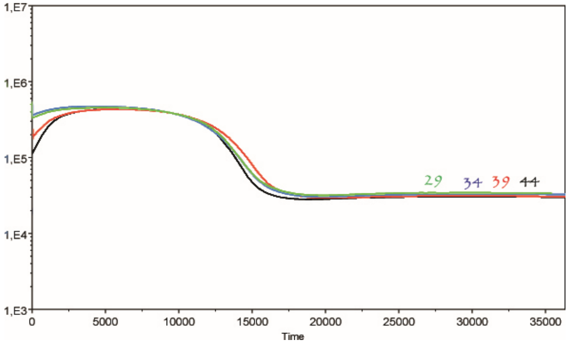

Nahua_at

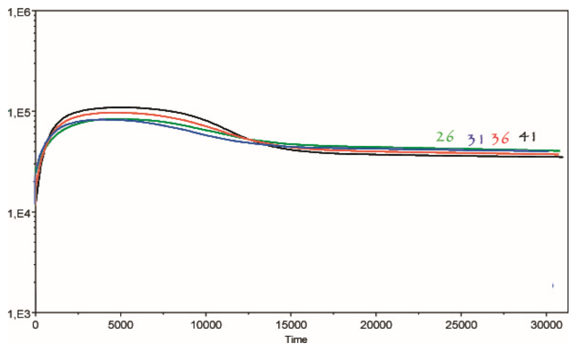

Mazateco

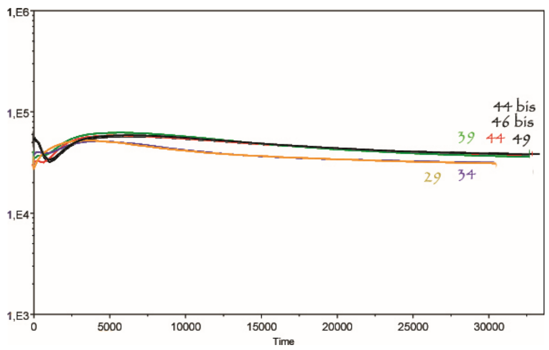

Mixe

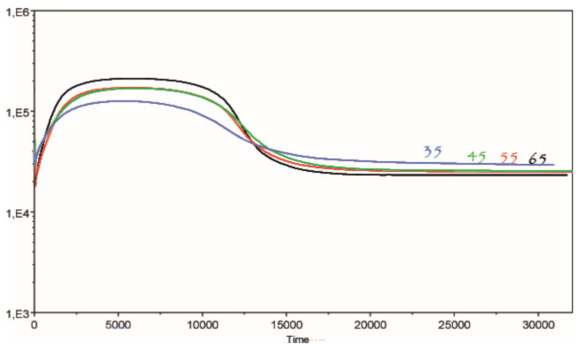

Mixteco

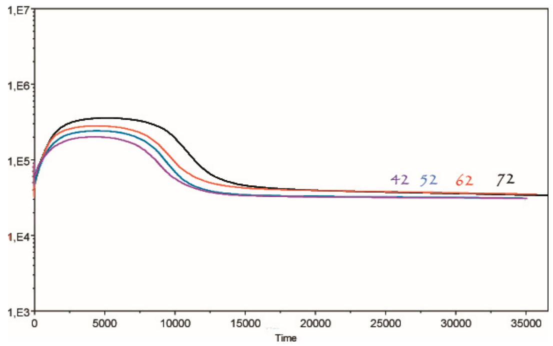

Zapoteco

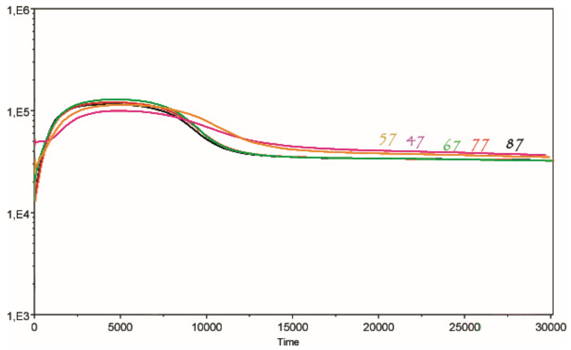

Tzotzil

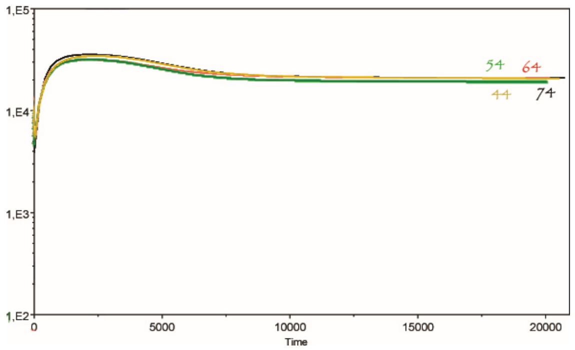

Tojolobal

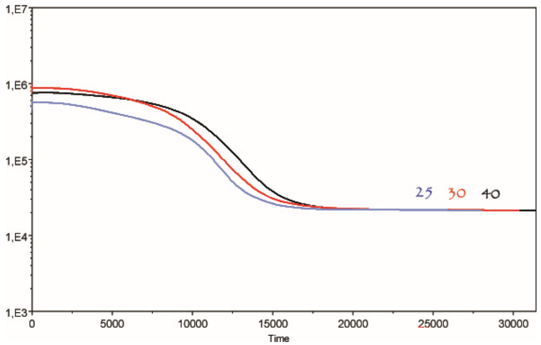

Maya_y

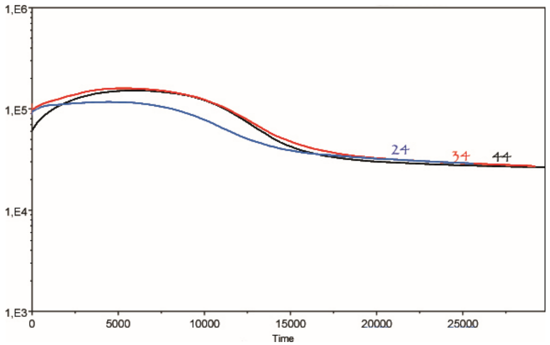

Maya_a

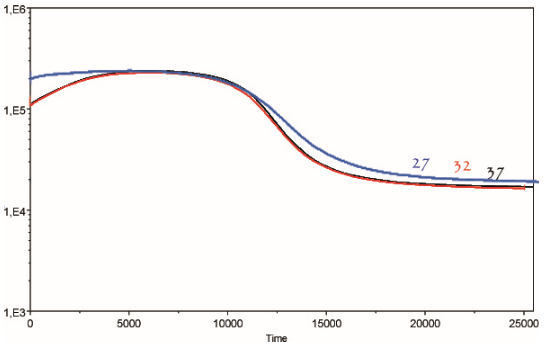

Maya_c

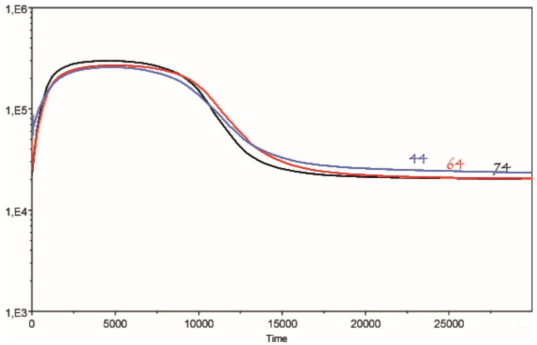

Maya_qr

Figure A. Bayesian skyline plot representing the 28 studied indigenous populations. A different number of samples have been selected at random in order to determine that sample size has no effect on the demographic profile.

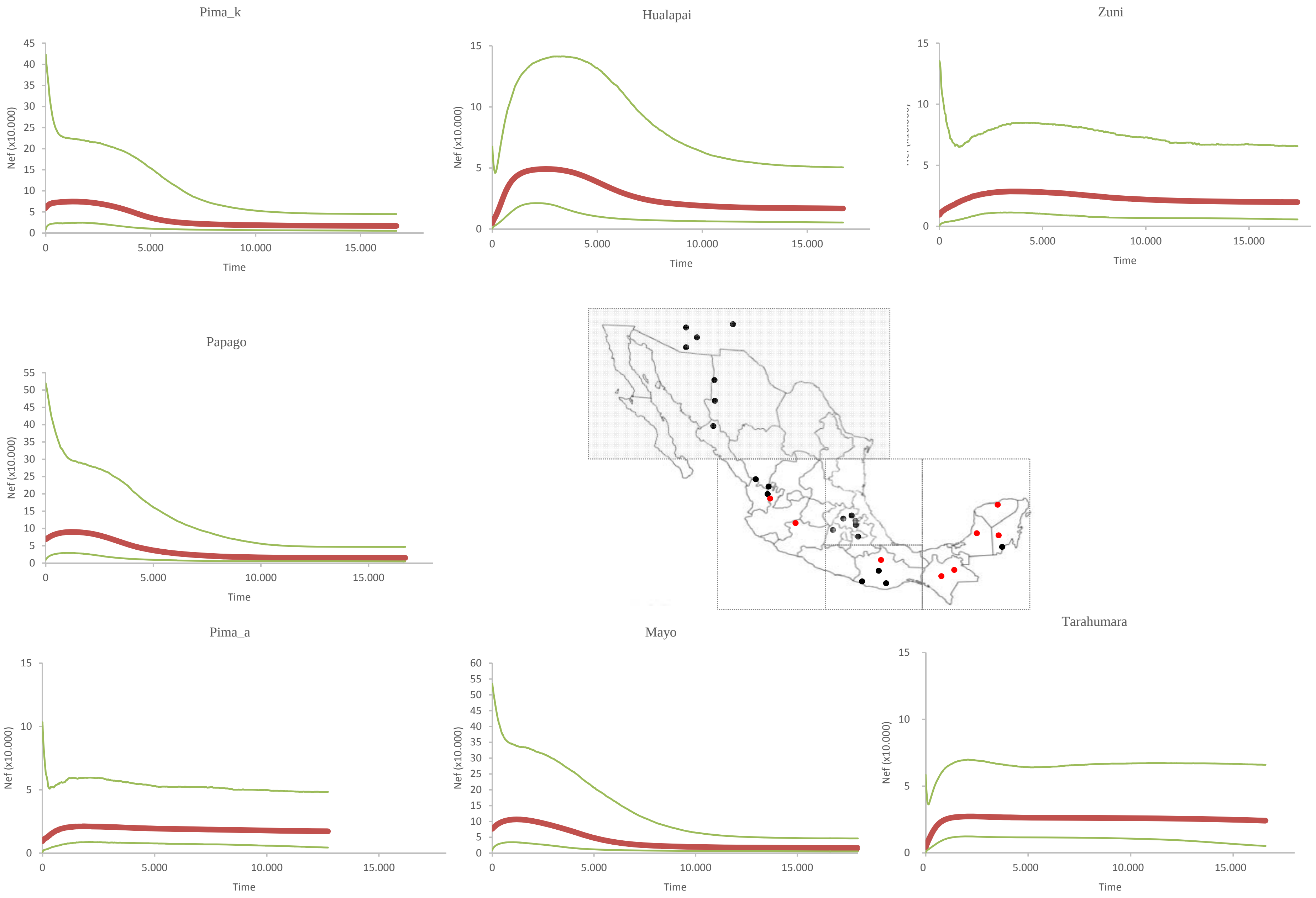
Pima_k
Nef (x10.000)
45
40
35
30
25
20
15
10
5
0
0
5.000
10.000
15.000
Time
Hualapai
Nef (x10.000)
15
10
5
0
0
5.000
10.000
15.000
Time
Zuni
15
10
5
0
0
5.000
10.000
15.000
Time
Papago
Nef (x10.000)
55
50
45
40
35
30
25
20
15
10
5
0
0
5.000
10.000
15.000
Time
Pima_a
Nef (x10.000)
15
10
5
0
0
5.000
10.000
15.000
Time
Mayo
Nef (x10.000)
60
55
50
45
40
35
30
25
20
15
10
5
0
0
5.000
10.000
15.000
Time
Tarahumara
Nef (x10.000)
15
10
5
0
0
5.000
10.000
15.000
Time

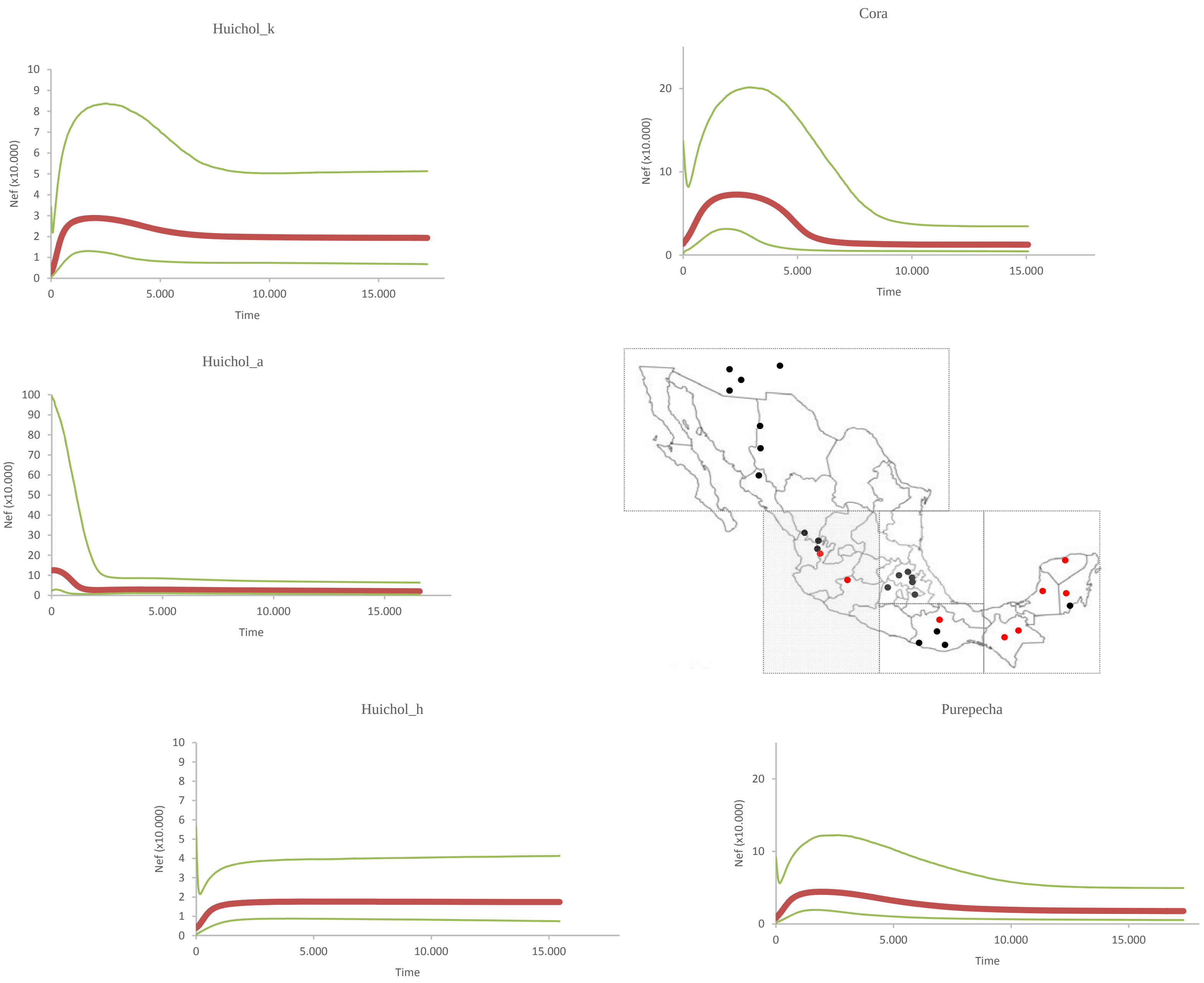
Huichol_k
Nef (x10.000)
10
9
8
7
6
5
4
3
2
1
0
0
5.000
10.000
15.000
Time
Cora
Nef (x10.000)
20
10
0
0
5.000
10.000
15.000
Time
Huichol_a
Nef (x10.000)
100
90
80
70
60
50
40
30
20
10
0
0
5.000
10.000
15.000
Time
Huichol_h
Nef (x10.000)
10
9
8
7
6
5
4
3
2
1
0
0
5.000
10.000
15.000
Time
Purepecha
Nef (x10.000)
20
10
0
0
5.000
10.000
15.000
Time

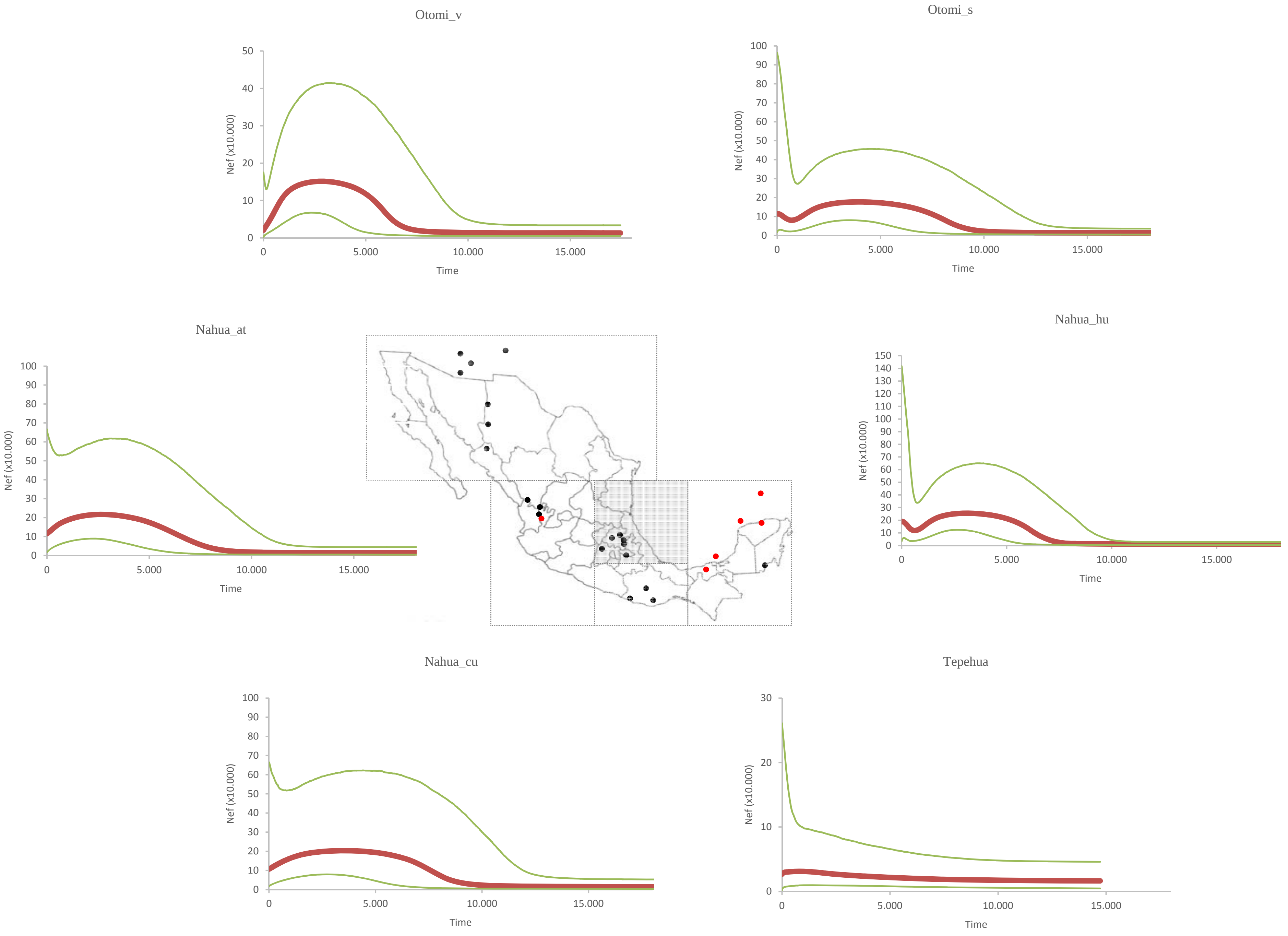
Otomi_v
Nef (x10.000)
50
40
30
20
10
0
0
5.000
10.000
15.000
Time
Otomi_s
Nef (x10.000)
100
90
80
70
60
50
40
30
20
10
0
0
5.000
10.000
15.000
Time
Nahua_at
Nef (x10.000)
100
90
80
70
60
50
40
30
20
10
0
0
5.000
10.000
15.000
Time
Nahua_hu
Nef (x10.000)
150
140
130
120
110
100
90
80
70
60
50
40
30
20
10
0
0
5.000
10.000
15.000
Time
Nahua_cu
Nef (x10.000)
100
90
80
70
60
50
40
30
20
10
0
0
5.000
10.000
15.000
Time
Tepehua
Nef (x10.000)
30
20
10
0
0
5.000
10.000
15.000
Time

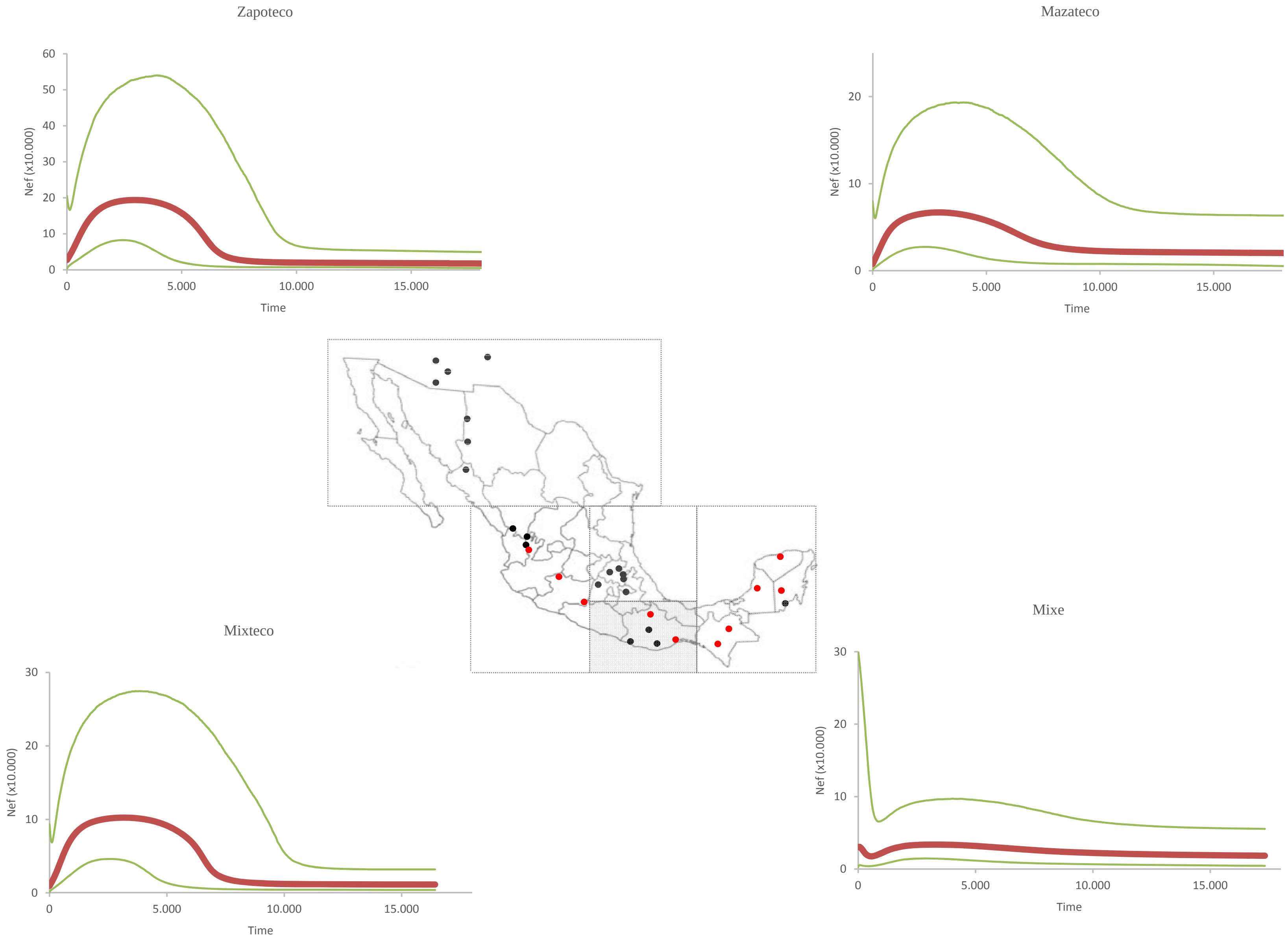

Zapoteco
60
50
40
30
20
10
0
Nef (x10.000)
0
5.000
10.000
15.000
Time
Mazateco
20
10
0
Nef (x10.000)
0
5.000
10.000
15.000
Time
Mixteco
30
20
10
0
Nef (x10.000)
0
5.000
10.000
15.000
Time
Mixe
30
20
10
0
Nef (x10.000)
0
5.000
10.000
15.000
Time

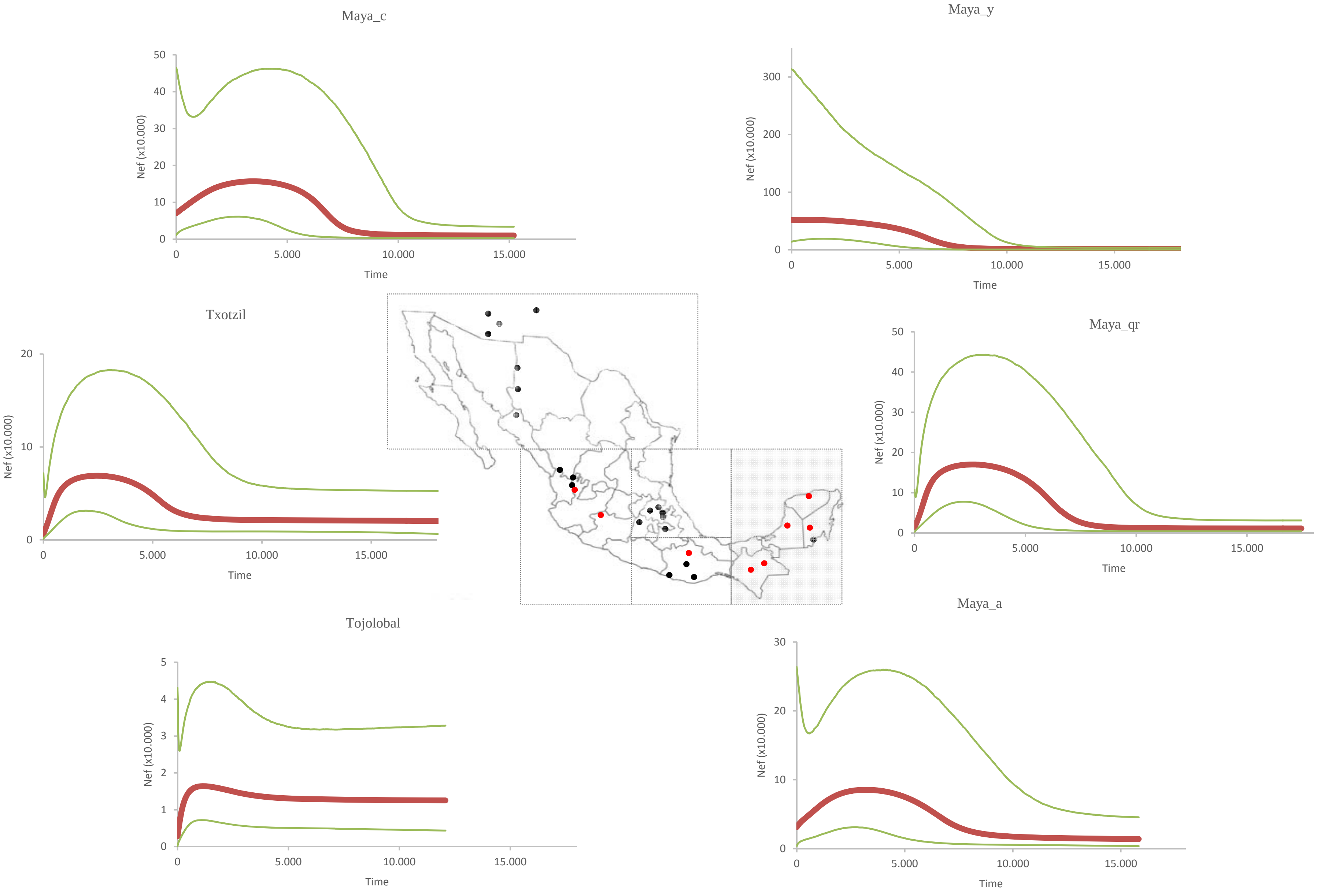

Figure B. The 28 studied indigenous populations grouped by cultural areas. The Nef median value is represented in the Bayesian skyline plot including confidence intervals.

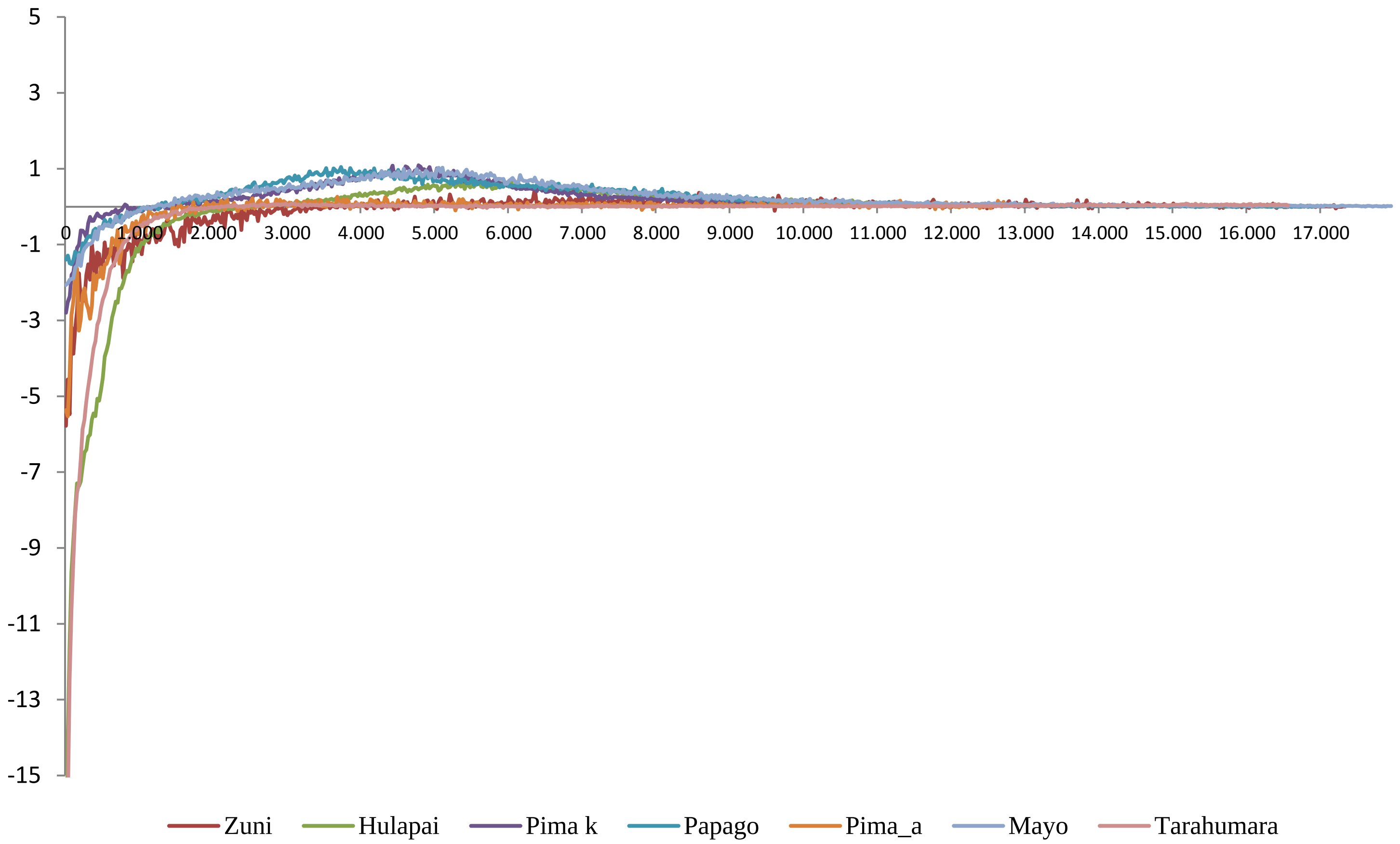

5
3
1
-1
-3
-5
-7
-9
-11
-13
-15
0
1.000
2.000
3.000
4.000
5.000
6.000
7.000
8.000
9.000
10.000
11.000
12.000
13.000
14.000
15.000
16.000
17.000
Zuni
Hulapai
Pima k
Papago
Pima_a
Mayo
Tarahumara

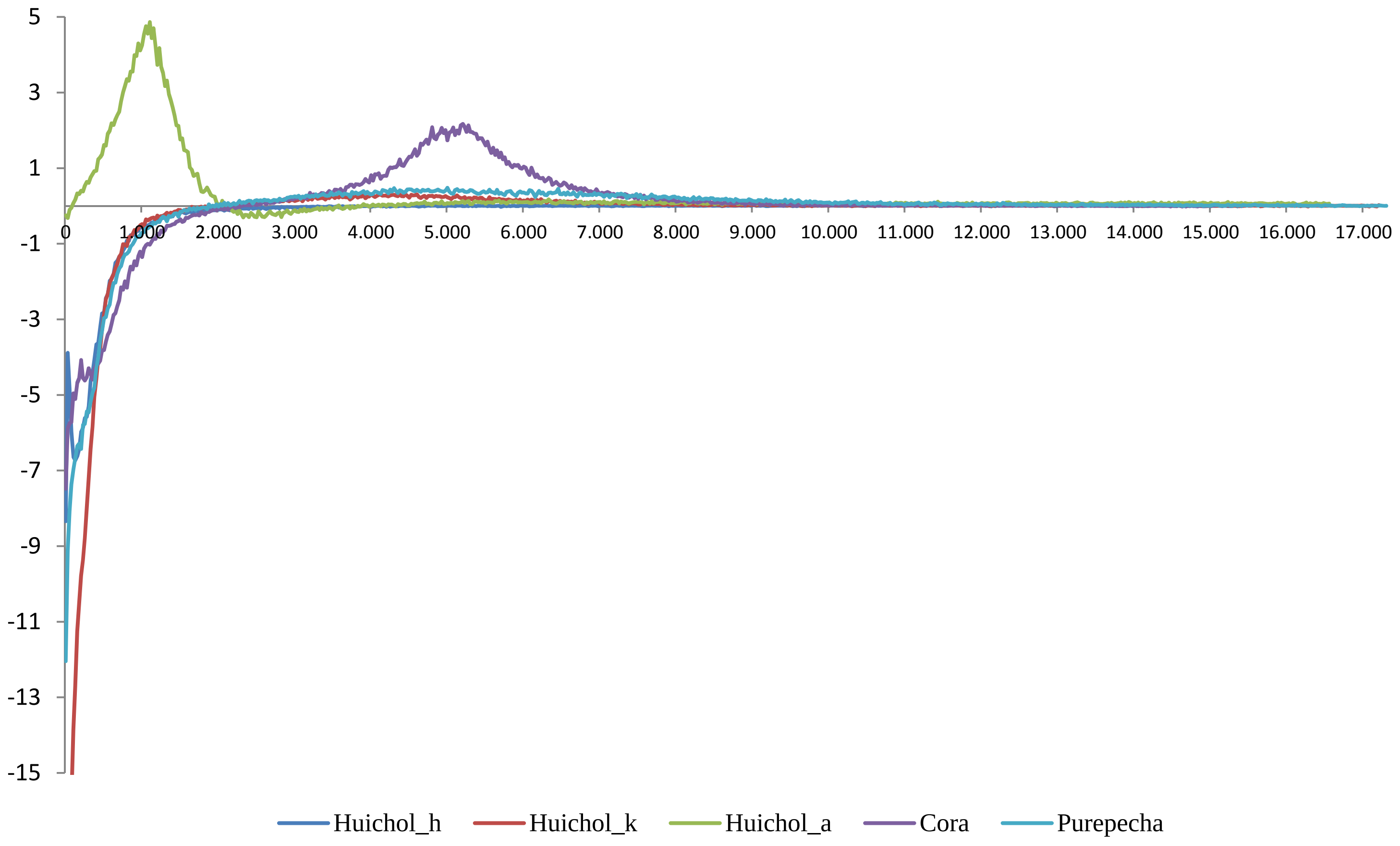
5
3
1
-1
-3
-5
-7
-9
-11
-13
-15
0
1.000
2.000
3.000
4.000
5.000
6.000
7.000
8.000
9.000
10.000
11.000
12.000
13.000
14.000
15.000
16.000
17.000
Huichol_h
Huichol_k
Huichol_a
Cora
Purepecha

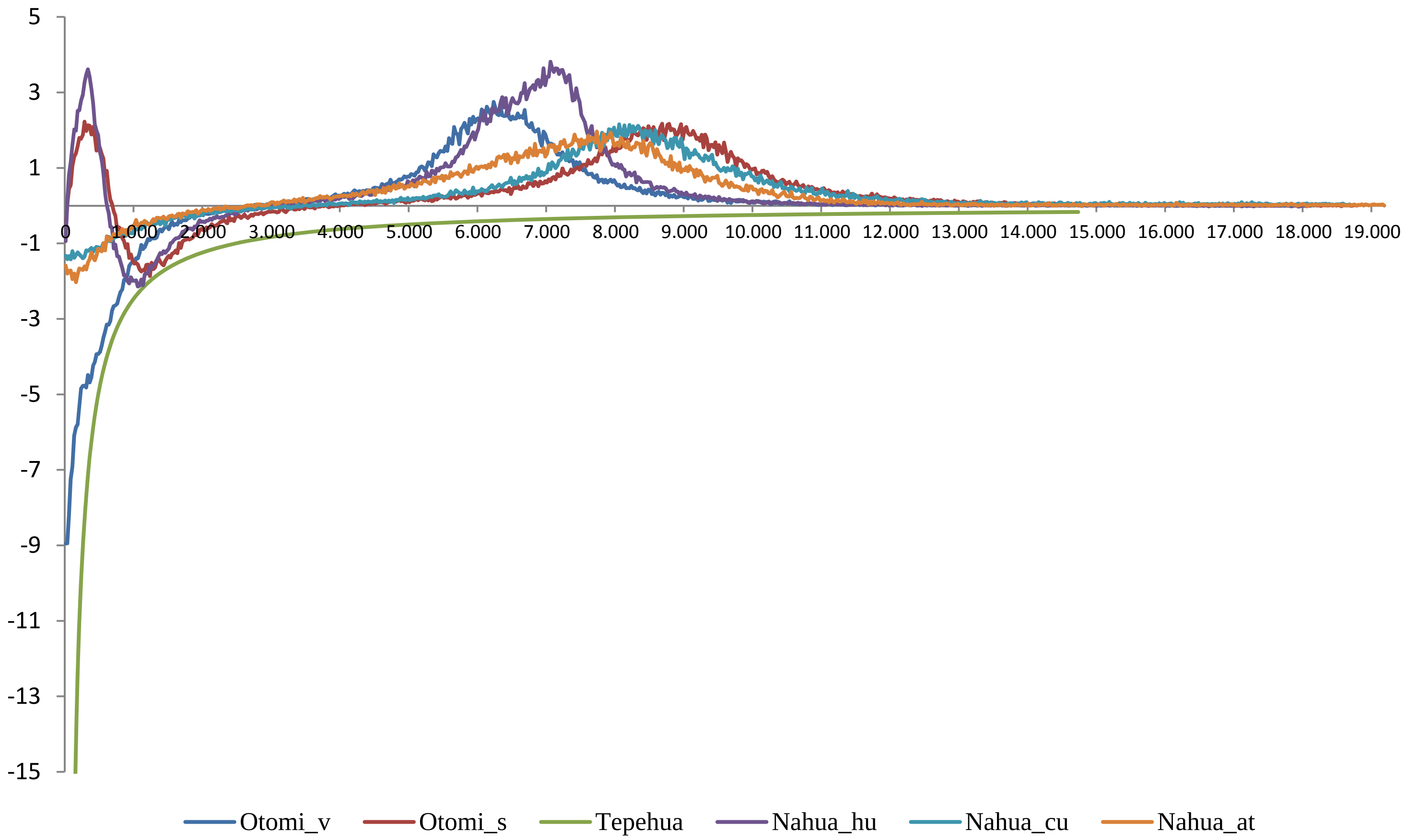
5
3
1
-1
-3
-5
-7
-9
-11
-13
-15
0
1.000
2.000
3.000
4.000
5.000
6.000
7.000
8.000
9.000
10.000
11.000
12.000
13.000
14.000
15.000
16.000
17.000
18.000
19.000
Otomi_v
Otomi_s
Tepehua
Nahua_hu
Nahua_cu
Nahua_at

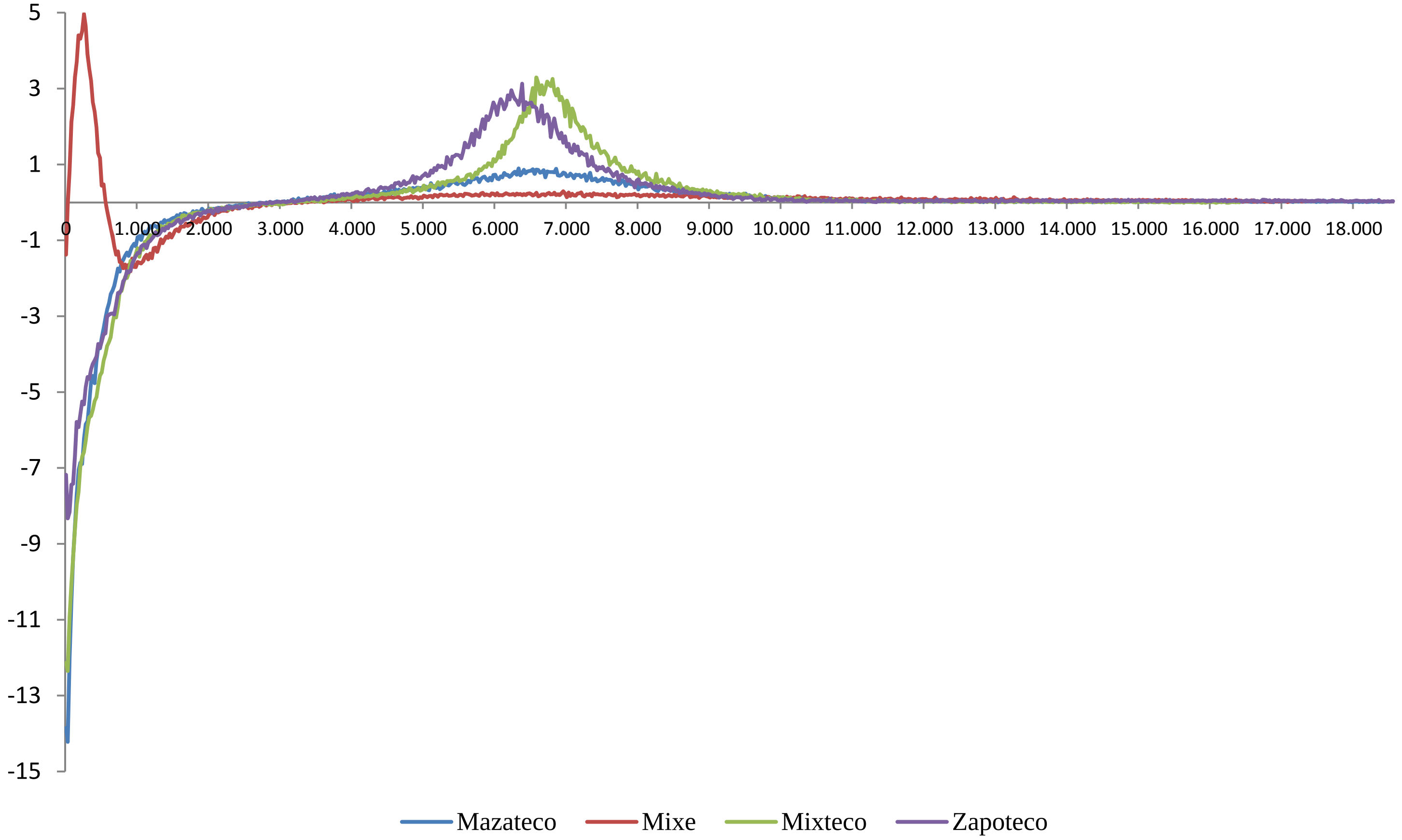

5
3
1
-1
-3
-5
-7
-9
-11
-13
-15
0
1.000
2.000
3.000
4.000
5.000
6.000
7.000
8.000
9.000
10.000
11.000
12.000
13.000
14.000
15.000
16.000
17.000
18.000
Mazateco
Mixe
Mixteco
Zapoteco

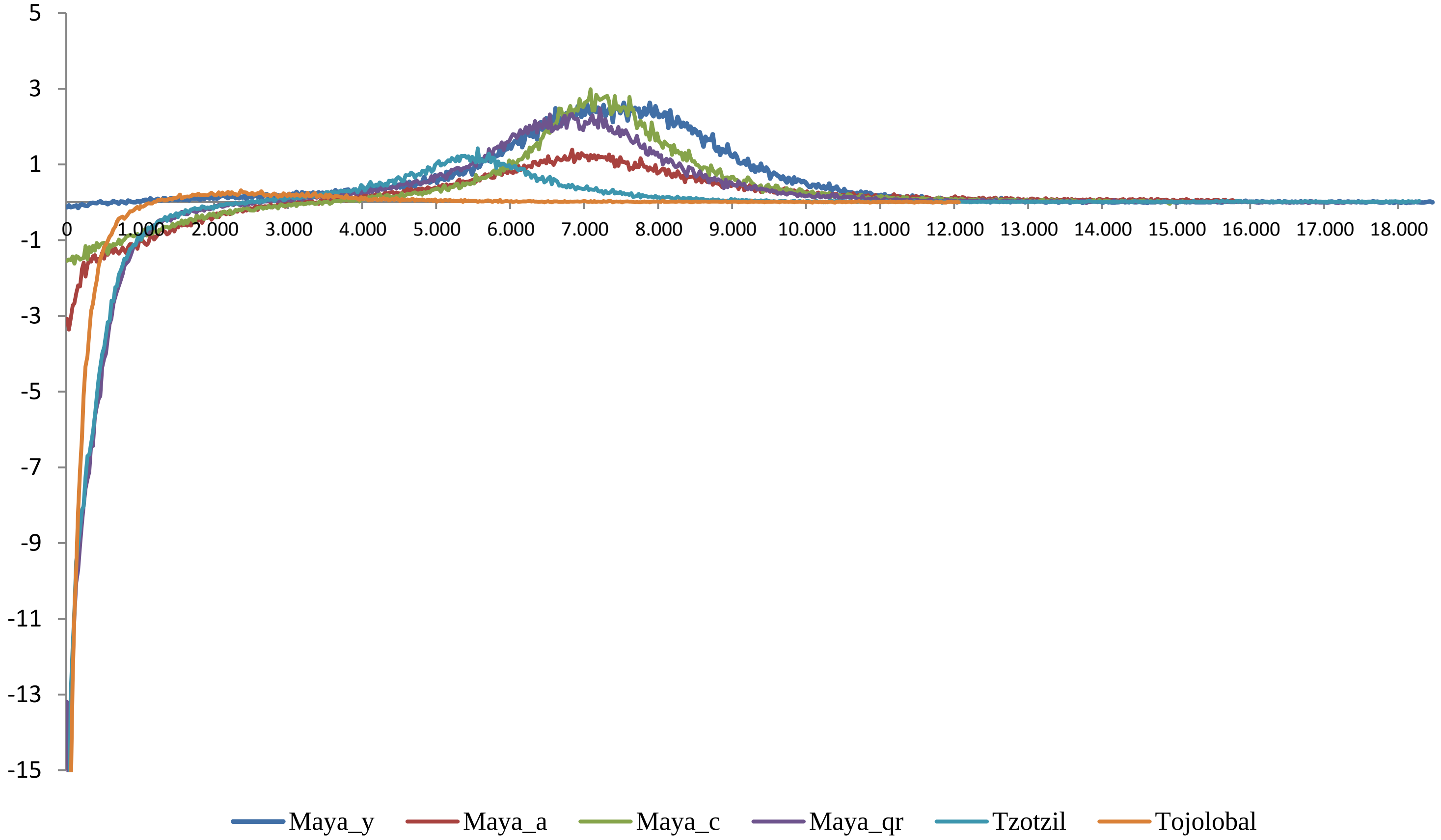

Figure C. IGR values temporary evolution for each indigenous group grouped by cultural areas. The Y-axis represents IGR percentage value and X-axis time in ybp.

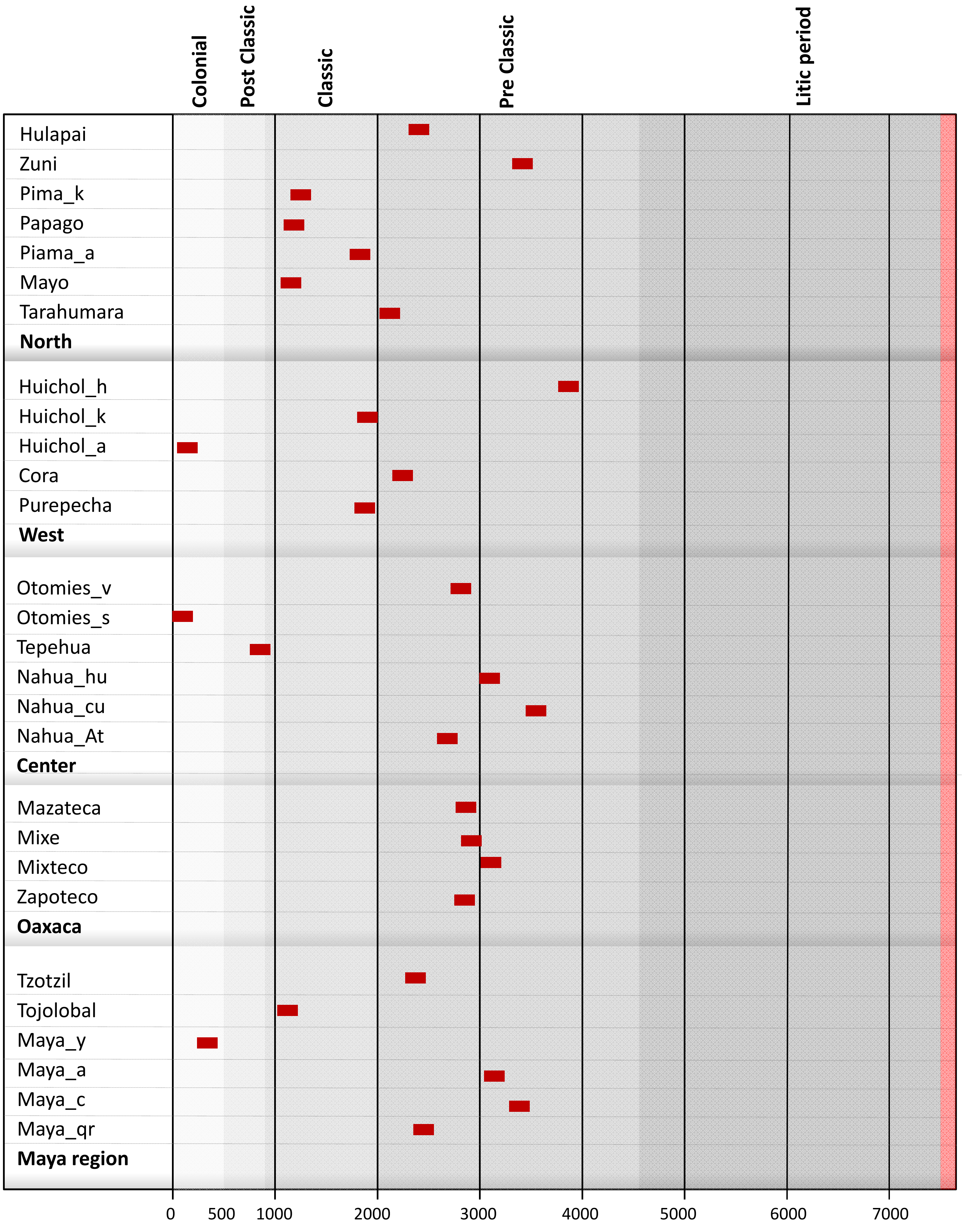

Figure D. Temporary distribution (bottom) according to Mesoamerican periods (top) in which the trend inversion occurred. The vertical red stripe indicates periods previous to 8,000 ybp.
